# Influence of weather variables on population dynamics of rice insect pests and their natural enemies: A six year light trap study in Gazipur, Bangladesh

**DOI:** 10.1371/journal.pone.0358423

**Published:** 2026-10-07

**Authors:** Tapon Kumar Roy, Mir Md Moniruzzaman Kabir, Md Nazmul Bari, Md Mosaddeque Hossain, Anamika Sannal

**Affiliations:** 1 Entomology Division, Bangladesh Rice Research Institute (BRRI), Gazipur, Bangladesh; 2 Plant Pathology Division, Gazipur Agricultural University (GAU), Gazipur, Bangladesh; Chulalongkorn University, THAILAND

## Abstract

Sustainable rice production in tropical and subtropical agroecosystems, it is essential to know, how climatic variability regulates the dynamics of insect pests and natural enemies. Our study evaluated the monthly and seasonal pattern of key weather variables and their influence on the abundance and interaction of major insect pests and their natural enemies from January 2019 to December 2024 through light trap catch in Gazipur, Bangladesh. Temperature was highest from March to October (maximum:32.87–35.25°C; mean:26.58–30.31°C), peaking during the Aus season, while relative humidity and rainfall were maximum during the monsoon month (June-October). Two peaked abundance of planthoppers, leafhoppers, rice leaf folder, stem borer, caseworm, rice bug and cricket showed in September-November and April-May coinciding mainly in Aman season and rice transitional periods under reproductive phases of rice. The pressure of insect pests was comparatively lower during the Boro season. Natural enemies; spiders, carabid beetles, ladybird beetles, odonates, and staphylinid beetles displayed distinct seasonal and climatic responses, with peaked activity largely synchronized with abundance of insect pests. Correlation analysis disclosed that temperature, rainfall and relative humidity influenced pest and predator populations differently. Temperature positively correlated most of the pests and natural enemies except staphylinid beetles, green mirid bug and spiders. Significant positive correlations among most insect pests and selective predator-prey relationships were revealed, highlighting intricate interactions. These findings, manifested that rice pest dynamics are strongly formed by seasonal climate variability, crop growth, and cropping transitions period, emphasizing the importance of climate-based monitoring and ecologically based integrated pest management strategies under changing climatic scenario thus helps to reduce insecticide use.

## 1. Introduction

Rice (*Oryza sativa* L.) is the principle staple cereal food for more than half of the population of the world with largest cultivation, production and consumption are contributing by South and Southeast Asia like (China, India, Bangladesh, Vietnam, Thailand, Pakistan, Malaysia etc). Cultivation of rice in these region, is typically characterized by diverse agro-ecological conditions, multiple cropping seasons and strong dependence on monsoon driven climatic variability [1]. These climatic heterogeneity profoundly influences the growth of rice, dynamics of pest population as well as the activity of natural enemies. The stability and productivity of rice is crucial for ensuring food security, compliance sustainable global economic and social development. But the cultivation of rice mostly depends on biotic and abiotic constraint, variety, management practice and climatic variables.

In addition, rice agroecosystem occupies a diverse abundance of rice arthropod species, with insect pests and natural enemies. Among biotic constraints, the abundance of insect pests in rice fields remain a major threat to sustainable rice production and responsible for substantial yield losses. Sap feeding insects likes planthoppers and leafhoppers, internal feeders like stem borer and gall midge, foliage feeders includes leaf folder and caseworms and grain feeders like rice bug are particularly destructive under favorable environment conditions [2–5]. The dynamics of these insects population are highly sensitive to weather variables like temperature, rainfall and relative humidity, which are directly responsible for mating, reproduction, survival and dispersal [3,6,7]. Consequently, fluctuation of temperature often result in seasonal pest outbreaks, particularly during monsoon period and rice transitional phases.

There are more than one rice season in rice growing regions such as Bangladesh, where rice cultivation is experienced with three distinct seasons-Aus, Aman and Boro. Each season characterized by unique climatic variables, cropping calendars and management practice. The Aus and Aman seasons are generally coincide with higher temperatures, increase humidity and substantial rainfall, conversely Boro season is under comparatively cooler, drier month and irrigated conditions. These seasonal contrasts create distinct ecological distribution which influence pest abundance, species composition and outbreak potential [4,8,9]. However, the pest pressure is not uniform throughout the seasons; rather, during transitional periods between crops it often intensifies, when residual host plants, overlapping of generations and changing microclimates facilitate rapid buildup of insect pests population.

Conversely, besides insect pests, rice fields support a diverse species of natural enemies including spiders, beetles, odonates and predatory bugs, which play a crucial role for maintaining ecological balance by suppressing pest populations [3,10,11]. Predatory species likes carabid beetle, staphylinid beetles, ladybird beetles, green mirid bug, spiders, dragonflies and damselflies are essential components of rice ecosystems, highlighting dynamically to both prey availability and environmental variables [3,5]. The abundance of natural enemies are strongly influenced by crop phenology, seasonal habitat suitability and weather variables, which may either enhance or disrupt predator-prey synchrony.

The interaction between insect pests and natural enemies with weather variables are intricate and often species specific. Temporal variables can accelerate pest reproduction and development while simultaneously enhancing or constraining predator activity, whereas relative humidity and rainfall may favor foliage feeders but negatively impact predator foraging efficiency or survival [12]. Despite growing acknowledge these interaction, majority of current research focuses on individual pest species of single seasons, offering little insight into the integrated dynamics of insect pests and natural enemy population across multiple cropping seasons and climatic variables. But, insect population behavior and trophic interactions in rice ecosystems are further modify due to altered rainfall patterns, increasing temperature variability and shifting monsoon dynamics in the context of climate change [3,6,13]. Therefore, proper understanding the abundance of insect pests and natural enemies according to weather variable and seasons is therefore critical for development and forecasting of climate-resilient and ecologically based integrated pest management strategies.

Based on this backdrop, our present study aimed to characterize the monthly and seasonal variations in key weather variables, identify and quantify the monthly, seasonal and transitional abundance of major insect pests and their natural enemies; and elucidate the influence of temporal, rainfall, relative humidity on insect-natural enemy interactions in rice based agroecosystem caught by light trap. We hypothesized that, i) the abundance of major rice insect pests and natural enemies differs significantly among rice growing seasons; ii) weather variables, particularly temperature, rainfall and relative humidity significantly influence the temporal dynamics of insect pests and natural enemies; and iii) predator abundance is associated with prey availability.

## 2. Materials and methods

### 2.1. Study area

The study was conducted at the Bangladesh Rice Research Institute (BRRI), Gazipur (Latitude: 23.99035; Longitude: 90.40433) Bangladesh research field site. A total 76.82 ha of land of BRRI including office and research field.

### 2.2. Scenario of weather of the study area and crop management practice

The study area was experienced with tropical rainfall between April and October, while remaining month felt scattered rainfall. The study area also experienced with hot, humid and cold weather conditions. Additionally, from May to September the study area experienced with the hot and humid weather and from November to February the cold weather observed. Monthly weather data on temperatures (maximum, minimum), relative humidity (%), and total rainfall were documented from January 2019 to December 2024 (the study period) and presented in the Table 2.

Weather data were collected from the “Davis automatic weather station” operated by Entomology division located at the research field of Bangladesh Rice Research Institute (BRRI), Gazipur. The station continuously recorded daily maximum, minimum and mean temperature (°C), rainfall (mm), relative humidity (%), sunshine duration using calibrated meteorological sensors. From which daily observations were recorded data in every one hour. Daily observations were aggregated into monthly means (cumulative monthly rainfall) for analysis. All weather records were checked for completeness and consistency before analysis. Missing values were rare (<1%) and were replaced using another “Davis automated weather station” data and no missing metrological values were included in the final dataset used for analysis.

Normally seedlings were grown in seedbed and then transplanted in the main rice fields. The rice fields was fertilized uniformly with nitrogen, phosphorus, K_2_O, gypsum and zinc sulfate @130–150, 50, 80–90, 45 and 2.0 kg ha^-1^ respectively during the boro season, @80–90, 25–30, 60–65, 25, and 1 kg ha^-1^ during the aman season and @75, 20–25, 50, 20 and 1 kg ha^-1^ during the aus season, respectively. The total amount of “triple super phosphate”, gypsum, “zinc sulphate” and two-third of “muriate of potash” were applied during the final land preparation time. One third of urea fertilizer was applied as basal dose at 10 days after transplanting (DAT), the remaining urea fertilizer then applied as top dressed in two equal split doses at 25–30 DAT and 40–45DAT. The remaining “muriate of potash” was applied during third top dressed of urea time. For the management of weeds normally manual weeding done as per requirement. Irrigation was done as per requirement until flowering stage of rice plant. Alternate wetting and drying also practiced during the vegetative growth stage of rice. For the management of fungal diseases normally 1–2 times applications of fungicides and for insects 1–2 times applications of insecticide were sprayed as per requirement based on economic threshold levels.

### 2.3. Data collection

The study was operated daily from January 2019 and continued till December 2024 through regular collections of light trap insects. The study covered 2192 calendar days (trap nights) over the six year monitoring periods. A light trap (Pennsylvania light trap) was set up near the rice research fields to monitor the rice insect pest population on a daily basis. The trap used a 100-W tungsten light bulb with a metal framework that was positioned at a height of two meters above the ground. The lighting hour of the trap were set up from dusk till dawn and day length varied from 10 to 12 hours per night based on seasonal photoperiod. A collecting cage was attached below the light trap to collect insects, which was a net bag (mesh number 40) was set up to quickly collect and eliminate all insects, even the smallest ones. The collection of both insect pests and natural enemies that had been trapped in the cages, in every morning. Following collection, the insects were then stored in freezer (−20°C) to ensure dead and after-then identification and counting the individual insects was done by using microscope and naked eye. The taxonomic characteristic of trapped insect pests were determined in accordance with Heinriches [14] methods and identification was done by following the same procedures throughout the study periods There were no other light source visible in the vicinity of the trap, and ensure other lights source at least 200 m distance from trapped light system. The trap was functioned throughout the study period, and there were no prolonged interruptions equipment failure or maintenance excluding bulb replacement. The bulb were replaced as per requirement. Furthermore, for large catch of insects, no subsampled was done and the accuracy of the trapped insects were confirmed by twice counting.

For the estimation of “Shannon Weiner diversity index” and “Pielou’s Evenness Index” following formula were used-

Shannon Weiner diversity index, H´=-$\sum_{i=\text{1}}^{S}\left(Pi.\text{ln}(Pi)\right)$

(Where, H is the Shannon-Wiener diversity index.

S is the total number of species in the community.

Pi is the proportion of individuals belonging to the i^th^ species relative to the total number of individuals.)

Maximum potential diversity: H_max_ =ln(S)

(Where, H_max_ is the maximum diversity.

ln is the natural logarithm.

S is the total number of species in the community)

Pielou’s Evenness Index (E): E=$\frac{H^{,}}{\text{ln}(S)}$

(Where, E is Pielou’s Evenness Index.

H´ is the Shannon-Wiener diversity index.

S is the total number of species.)

In Bangladesh, the rice cultivation in a year divided into three seasons; Aman, Boro and Aus (Table 1). So, when organizing the data, these rice growing seasons and growth stage of rice were taken into consideration for the season-wise dynamics study. But, there were overlapping of the rice growing seasons (Table 1). The Aus season coincides with both Aman and Boro seasons. There were both major and minor abundances of different types of rice insects and pests due to seasonal fluctuations. So, for overcome that problem we used a triple venn diagram to find out the quantity of arthropod natural enemies and insect pests by season.

**Table 1:** Rice growing seasons and growing stage of rice according to month in Bangladesh.

SL	Season	Month	Growth phase		
			Seedling	Tillering	Booting to heading
1	Boro (Irrigated)	November-May	Mid November-January	February-March	March-April
2	Aus (Monsoon)	April-August	April -May	May-June	June-July
3	Aman (Rainfed)	July-November	July-August	August-September	September-October

### 2.4. Statistical analysis

The daily trap night (trap night^-1^) catch was considered the basic observational unit. Daily insect counts were summed to obtain monthly catches. Furthermore, monthly values were subsequently grouped according to the three rice growing seasons (Boro, Aus and Aman) for seasonal analyses. The collected characteristics of a meteorological data from weather station of entomology division, at Bangladesh Rice Research Institute (BRRI), Gazipur were summarized using descriptive statistics. All statistical analyses were performed using R version 4.5.1 within the R studio (version 2025.09.2 + 418). For the normality test of the distribution of data we used “Shapiro-wilk normality test”. The distribution of each monthly variable was initially examined using statistics, including mean, variance, range and frequency distribution.

A negative binomial “generalized linear mixed model (GLMM)” including year as a random intercept was initially evaluated to account for year variability. However, the estimated variance of the year random effect was effectively zero and the model exhibited a singular fit. The simpler negative binomial “generalized linear model (GLM)” therefore provided an adequate and more appropriate representation of the data. The variance to mean relationship was also examined to identify potential over-dispersion due to the response variables consisted of non-negative integer counts and were frequently right skewed, “generalized linear models (GLMs)” with a log link were used. The monthly abundance of insect pests and natural enemies was analyzed using “generalized linear modelling” approach. “Poisson” GLMs were initially fitted to each taxon. Model adequacy was evaluated using simulation based residual diagnostics, including tests for dispersion and zero inflation. When significant over-dispersion was detected, a “negative binomial GLM” was fitted using *glm.nb ()* function in the “MASS (v7.3-66). Negative binomial GLM models were retained when they provided an appropriate variance structure and satisfactory residual diagnostics. For significant overall effects, estimated marginal means were obtained using “emmeans (v2.0.4) package and presented on the original response scale by back transformation from logarithmic link. Pairwise comparisons among factor levels were performed using multiplicity adjusted contrasts. The “Sidak” adjustment was used when family-wise error rate across the specified set of pairwise comparisons. Compact letter displays were generated using the *cld ()* function of the “multcomp (v1.4-31)” package. We used “DHARMa (v0.5.0)” package for model diagnostics.

Data visualization was conducted using “ggplot2 (v4.0.3)” package within the “tidyverse (v2.0.0)” framework. “Box and whisker” plots and “violin” plot was generated to illustrate the monthly meteorological variables, enabling visualization of data distribution, variability, median values, interquartile ranges and potential outliers. Data reshaping and graphical customization were performed using RColorBrewer (v1.1.-3); reshape2 (v1.4.4)” package. Three ‘Venn diagram’ categories were utilized to show the population throughout the year while considering the overlapping seasons using VenDiagram (v1.8.2). Venn diagrams were used descriptively to illustrate the shared and unique occurrence of insect taxa among observations classified within the three rice production seasons; they were not used to test statistically independent differences among seasons. Pearson correlation coefficients between insect populations and meteorological variables were calculated and visualized using the “metan (v1.19.0)” package in R studio (version 2025.09.2 + 418).

## 3. Results

### 3.1. Status of the weather variables according to month

The highest maximum temperature was demonstrated in April. The consecutive maximum temperate was found in the May, June and September month (Table 2). Comparatively, from March to October showed higher maximum temperature and at that time the maximum temperature exceed 32°C (Table 2). Similarly, from March to October demonstrated higher minimum temperature but peaked in July (27.09°C). March to October month also demonstrated the highest mean temperature but peaked in July. Furthermore, January (maximum 25.40°C, minimum 13.51°C and mean 19.46°C) was the coldest month of our study period and comparatively lowest temperature were exhibited from November to February (Table 2). The higher relative humidity (%) were observed from June to October month. The heavy rainfall occurred from May to October in our study period, but June and August found peaked rainfall month (Table 2).

**Table 2:** Weather scenario of study area according to month from January 2019 to December 2024.

Month	Maximum temperature (°C)	Minimum temperature (°C)	Mean temperature (°C)	Relative humidity (%)	Mean monthly total Rainfall (mm month^-1^)
January	25.40	13.51	19.46	71.20	6.67
February	28.73	15.60	22.17	65.12	9.44
March	32.87	20.29	26.58	61.81	33.69
April	35.25	24.22	29.74	65.65	23.97
May	34.66	24.95	29.80	71.91	122.42
June	33.71	26.54	30.13	79.28	168.17
July	33.52	27.09	30.31	79.61	97.09
August	33.25	26.80	30.03	79.82	157.63
September	33.82	26.68	30.25	77.68	105.68
October	33.15	24.99	29.07	73.54	122.08
November	30.93	19.97	25.45	65.83	9.16
December	26.59	15.76	21.18	67.69	13.97

### 3.2. Status of the weather variables according to rice growing seasons

The seasonal maximum temperature, minimum temperature and mean temperature exhibited highest in Aus season (34.08; 25.92; 30.00°C), followed by Aman (32.93; 25.11; 29.02°C) season and then Boro (30.58; 19.06; 24.82°C) season (Fig 1). The Aman (75.30%) and Aus (75.25%) seasons exhibited highest relative humidity (%) and followed by Boro (67.23%) season. Higher variability of rainfall was exhibited in both Aus, Aman and Boro seasons during the reporting periods. The Aus (113.85 mm season^-1^) season demonstrated the highest rainfall, which was followed by Aman (98.33 mm season^-1^) and Boro (35.03 mm season^-1^) seasons (Fig 1).

**Fig 1:**
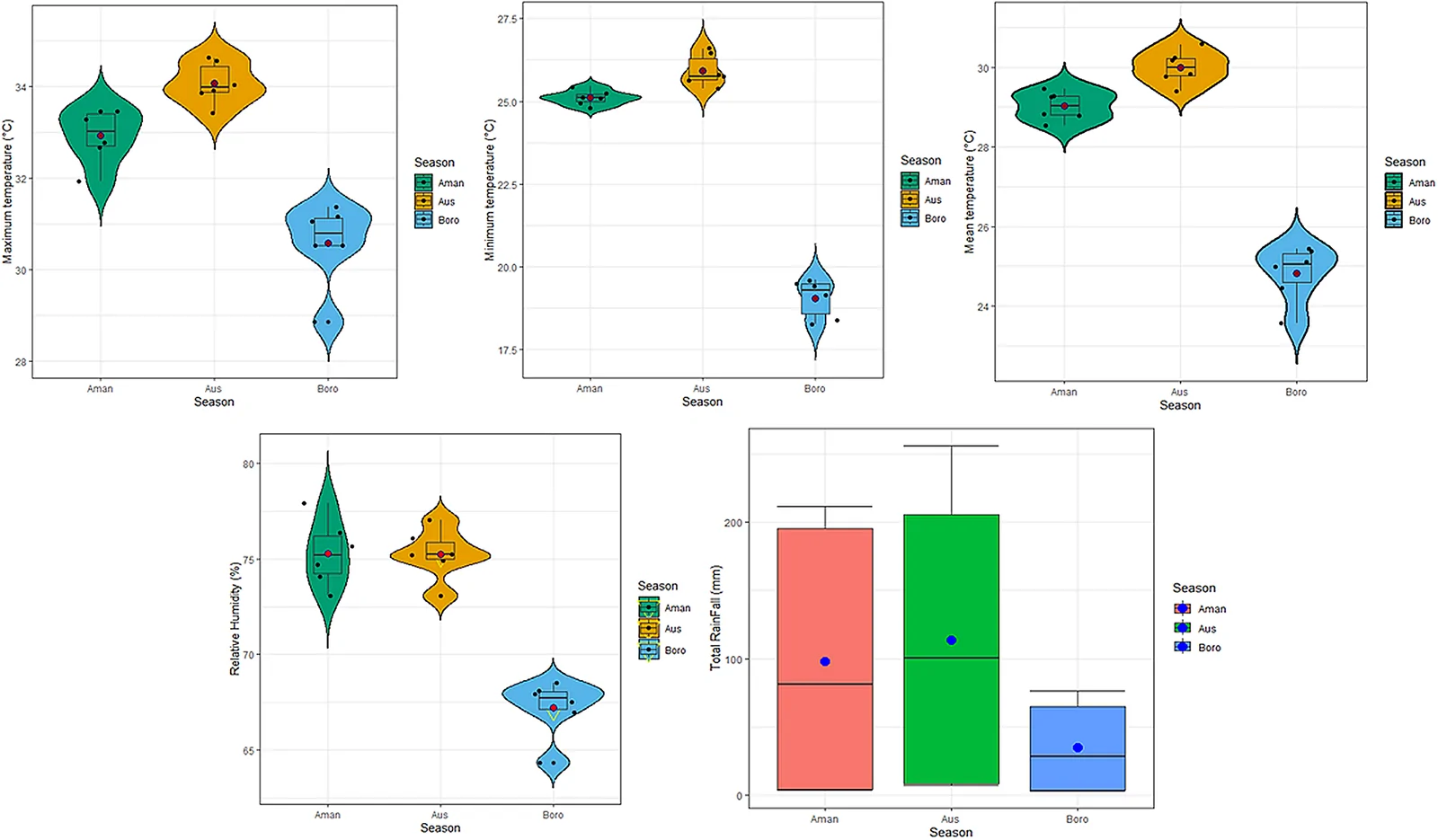
Average seasonal climatic scenario from 2019 to 2024 in Boro (irrigated), Aus (pre-monsoon) and Aman (monsoon), season in BRRI, Gazipur Bangladesh.

### 3.3. Comparative diversity of insect pests and natural enemy

The estimated Shannon-Wiener diversity index (H´) was 2.16 (‘-’sign neglected), which revealed a moderately high diversity insect pests and natural enemies, representing approximately 74.86% of the theoretical maximum diversity (Hmax = 2.89). The pielou’s evenness index (E = 0.75) indicated a relatively even distribution of individuals among the recorded species, although few species were considerably more abundant than others (Table 3). A total of 18 insect taxa, comprising 11 pest species and 7 natural enemy groups, were recorded during the study. Among the pest species, the brown planthopper was the most dominant species, accounting 34.69% of the total arthropod population (Pi = 0.3469) and contributed the largest proportion to the Shannon diversity index (Pi × lnPi = −0.3673). The green leafhopper representing 12.67% of the total population, followed by white-backed planthopper (10.84%), yellow stem borer (6.79%) and white leafhopper (6.59%).other pest species occurred comparatively lower frequencies. Natural enemies comprised a smaller proportion than herbivorous insects. Among the natural enemies, the staphylinid beetle was the most abundant (7.66%) followed by the greed mirid bug (6.14%) and the carabid beetle (3.30%).

**Table 3:** Population dynamics of insect and estimation of Shannon-Wiener diversity index, H_max_ and evenness of species.

Common name	Order name	Family name	Pi	Ln(Pi)	Pi*Ln(pi)
**Insect pests**					
Green leafhopper	Hemiptera	*Nephotettix virescens* (Distant)	0.1267	-2.0655	-0.2618
White leafhopper	Homoptera	*Cofana spectra* (Distant)	0.0659	-2.7203	-0.1791
Zigzag leafhopper	Hemiptera	*Maiestas dorsalis* (Motschulsky)	0.0396	-3.2287	-0.1279
Brown planthopper	Hemiptera	*Nilaparvata lugens* (Stål)	0.3469	-1.0589	-0.3673
White-backed planthopper	Hemiptera	*Sogatella furcifera* (Horvãth)	0.1084	-2.2222	-0.2408
Yellow stem borer	Lepidoptera	*Scirpophaga incertulas* (Walker)	0.0679	-2.6899	-0.1826
Rice leaffolder	Lepidoptera	*Cnaphalocrocis medinalis* (Guenée)	0.0088	-4.7297	-0.0418
Rice caseworm	Lepidoptera	*Parapoynx stag* (Zeller)	0.0105	-4.5553	-0.0479
Long horned cricket	Orthoptera	*Euscyrtus concinnus* (Haan)	0.0102	-4.5854	-0.0468
Field cricket	Orthoptera	*Tarbinskiellus portentosus* (Lichtenstein)	0.0124	-4.3862	-0.0546
Rice bug	Hemiptera	*Leptocorisa oratoria* (Fabricius)	0.0222	-3.8097	-0.0844
**Natural enemy**					
Carabid beetle	Coleoptera	*Ophionea nigrofasciata* (Schmidt-Gobel)	0.0330	-3.4110	-0.1126
Ladybird beetle	Coleoptera	*Coccinella septempunctata* (Linnaeus)	0.0087	-4.7498	-0.0411
Styphylinid beetle	Coleoptera	*Paederus fuscipes* (Fabricius)	0.0766	-2.5691	-0.1968
Green mirid bug	Hemiptera	*Cyrtorhinus lividipennis* (Reuter)	0.0614	-2.7901	-0.1714
Damselfly	Odonata	*Ischnura* spp	0.0003	-8.1704	-0.0023
Spider	Araneae	*Atypena formosana* (Oi)	0.0004	-7.8870	-0.0030
		*Oxyopes javanus* (Thorell)			
		*Tetragnatha virescens* (Okuma)			
		*Argiope catenulate* (Doleschall)			

Dragonfly	Odonata	*Diplacodes* spp (Kirby)	0.0002	-8.5172	-0.0017
H´=2.16		H_max_=2.89		E=0.75	

***Footnote***, H´: Shannon-Wiener diversity index; E: Pielou’s Evenness Index; H_max_: the maximum diversity; ln: the natural logarithm; Pi: is the proportion of individuals belonging to the i^th^ species relative to the total number of individuals.

### 3.4. Monthly abundance of insect pests

The abundance of all studied insect pests exhibited statistically significant difference according to month (Table 4, 5). The highest abundance of green leafhopper (GLH) was found in October (1547.0; 95% CI:1083–2209), followed by November, April and May respectively. The abundance of brown planthopper (BPH) was found maximum in November followed by October, December, April and May. Furthermore, the highest abundance of white-backed planthopper (WBPH) was exhibited in October followed by April, May, November and July (Table 4). The prevalence of yellow stem borer was found maximum in October and May, then followed by September and November. September and October were the month of highest abundance of rice leaf folder (RLF). The rice case worm abundance was found maximum in September, October and November (Table 5). November was the month of maximum rice bug abundance, followed by May and October. The abundance of long horned cricket was found maximum in October and November. May, June and July were the highest abundance month of field cricket followed by October and November. The abundance of white leafhopper (WLH) was found maximum in October followed by September and November. The abundance of zigzag leafhopper (ZzLH) was found maximum in November which was followed by October, and May (Table 5).

**Table 4:** GLM output for monthly abundance of insect pests from January 2019 to December 2024.

Month	Green leafhopper		Brown planthopper		White-backed planthopper		Yellow stem borer		Rice leaf folder	
	Estimated	95%CI	Estimated	95%CI	Estimated	95%CI	Estimated	95%CI	Estimated	95%CI
January	19.7 a	12.64-30.6	53.5 bc	33.47- 85.5	17.5 a	9.78- 31.3	110.8 ab	74.6- 165	24.2 bc	15.79- 36.99
February	12.2 a	7.47-19.8	26.8 ab	16.35- 44.0	17.0 a	9.48- 30.5	127.3 ab	85.9- 189	11.7 ab	7.14- 19.07
March	71.2 b	48.61- 104.2	227.8 d	145.58-356.6	272.8c-e	162.84- 457.1	136.2 ab	91.9- 202	19.2 bc	12.31- 29.84
April	448.2 d	312.83- 662.1	1550.5e	996.42- 2412.7	580.2 e	347.17- 969.5	174.7 bc	118.3- 258	21.8 bc	14.16- 33.65
May	430.3 d	300.33- 616.6	2076.50ef	1334.78- 3230.4	456.7 e	273.10- 763.6	341.8 d	232.6- 502	14.7 bc	9.20- 23.39
June	182.8 c	126.85- 263.5	399.5 d	256.0- 623.4	108.67 b	82.78- 183.4	98.2 ab	66.0- 146	5.0 a	2.66- 9.39
July	97.3 b	66.93- 141.5	16.2 a	9.53- 27.4	8.5 e	4.44- 16.3	79.7 a	53.3- 119	14.8 bc	9.31- 23.63
August	184.2 c	127.78- 265.4	106.7 c	67.65-168.2	139.3 bc	82.78- 234.5	118.7 ab	80.0- 176	24.3 bc	15.90- 37.23
September	385.2 d	268.67- 552.2	469.2 d	300.82- 731.7	67.3 b	39.61- 114.5	269.5 cd	183.1- 397	61.2 d	41.68- 89.76
October	1547.0 f	1083- 2209	2095.0 ef	1411.01- 3414.6	1540.8 f	923.35- 2571.3	416.7 d	283.8- 612	55.5 d	37.71- 81.69
November	822.8 e	575.46-1176.5	3395.7 f	2183.38- 5181.1	340.0 de	203.10- 569.1	332.0 cd	225.9- 488	22.7 bc	14.74- 34.85
December	130.0 bc	89.82- 188.1	1334.0 e	857.15- 2076.1	153.8 b-d	91.48- 258.7	114.0 ab	76.8- 169	26.7 c	17.53- 40.56

**Table 5:** GLM output for monthly abundance of insect pests from January 2019 to December 2024.

Month	Caseworm		Rice bug		Long horned cricket		Field cricket		White leafhopper		Zigzag leafhopper	
	Estimated	95%CI	Estimated	95%CI	Estimated	95%CI	Estimated	95%CI	Estimated	95%CI	Estimated	95%CI
January	19.2a-c	11.57- 31.8	7.33 ab	3.95- 13.62	4.33 bc	2.19- 8.61	2.00 a	0.82-4.86	3.17 a	1.50- 6.67	37.3bc	24.87- 56
February	16.3a-c	9.74- 27.4	2.83 a	1.24- 6.46	0.50 a	0.09- 2.73	2.83 a	1.31- 6.11	14.50 b	9.07-23.17	10.2 a	6.09- 17
March	26.5b-d	16.31- 43	2.67 a	1.15-6.19	0.17 ab	0.009- 2.98	6.33 a	3.58- 11.19	135.67 de	93.82-196.17	39.7 bc	26.50-59
April	24.2 bc	14.80- 39.5	39.83 cd	24.61- 64.47	37.0 e	23.83- 57.44	43.33 cd	29.77- 63.07	174.67e	121.16- 251.79	245.0 ef	169.88- 353
May	13.33 ab	7.81- 22.8	133.0 e	84.30- 209.85	14.0 d	8.46- 23.17	75.33 de	52.71- 107.67	83.50 cd	57.26- 121.76	180.3 de	124.71- 261
June	8.5 a	4.73- 15.3	54.33 d	33.89- 87.11	12.17 d	7.25- 20.43	81.50 de	57.13- 116.26	60.33 c	41.04-88.70	24.8 b	16.18-38
July	15.8a-c	9.42- 26.6	18.83 bc	11.20- 31.67	2.83 a-c	1.28- 6.29	73.67 de	51.51- 105.35	140.33de	97.10-202.83	0.00 a	–
August	32.3c-e	20.10- 52.0	28.33 cd	17.26- 46.52	2.33 a-c	0.98- 5.52	17.17 b	11.11- 26.51	180.17 e	125.02-259.64	44.5 bc	29.86-66
September	55.5 de	35.15- 87.6	27.17 cd	16.51- 44.69	6.0 c-d	3.23- 11.15	29.17 bc	19.65- 43.30	335.33 f	233.82-480.92	58.50 c	39.62-86
October	60.7 e	38.51- 95.6	153.33 e	97.33- 241.57	144.0 f	95.80- 216.46	36.00 c	24.53- 52.84	639.00 g	446.75-913.98	228.3 d-f	158.24-329
November	61.2 e	38.84- 96.3	256.33 e	163.35- 402.25	118.83 f	78.86- 179.07	51.83 c-e	35.86- 74.93	383.17 fg	267.36-549.14	355.5 f	247.09-511
December	25.7b-d	15.77- 41.8	33.0 cd	20.24- 53.80	6.33 cd	3.44- 11.65	6.17 a	3.47- 10.96	100.33 c-e	69.06-145.77	129.2 d	88.96-188

***Footnote***; values are estimated marginal means froma negative-binomial model with log link; CI: confidence interval; confident level used: 0.95; confidence level adjustment with “sidak method” for 12 estimates; significance level used, alpha = 0.05; Means followed by different lowercase letters within a column differ significantly. Means with the similar lowercase letter within a column do not differ significantly

### 3.5. Monthly abundance of natural enemies

Carabid beetle abundance differed significantly among moths. The highest abundance of carabid beetle (CBB) was found in November (289.3), followed by October and May, respectively. The ladybird beetle (LBB) abundance also showed monthly variation was found maximum in November (73.7 individuals; 95% CI: 54.0–100.4), whereas abundance remained relatively low from January to April and during July-September. March (781.8 individuals; 95% CI: 585.6–1043.9) was the month of highest abundance of staphylinid beetle (StPB), which was followed by a marked decline from April onward. November and December were the time of highest abundance of green mirid bug (GMB), which was followed by April (Table 6).

**Table 6:** GLM output for monthly abundance of arthropod natural enemies from January 2019 to December 2024.

Month	Carabid beetle		Ladybird beetle		Staphylinid beetle		Green mirid bug		Damselfly		Dragonfly		Spider	
	Estimated	95%CI	Estimated	95%CI	Estimated	95%CI	Estimated	95%CI	Estimated	95%CI	Estimated	95%CI	Estimated	95%CI
January	25.0 a	17.8- 35.2	12.8 a	8.36- 19.7	452.5 d	338.2- 605.5	22.3 c	14.9- 33.3	0.00 a	–	0.00 a	–	2.17 a	0.98- 5.0
February	38.3 ab	28.1- 52.4	17.0 a	11.43- 25.3	617.0 de	461.7- 824.5	0.3 a	0.04- 2.58	0.67 a	0.16- 3.0	0.00 a	–	1.00 a	0.31- 3.0
March	39.0 ab	28.6- 53.2	12.3 a	7.99- 19.0	781.8 e	585.6- 1043.9	12.2 bc	7.69- 19.26	0.67 a	0.16- 3.0	0.17 a	0.01- 3.0	0.50 a	0.10- 3.0
April	46.0 b	34.0- 62.3	15.3 a	10.20- 23.1	52.33 c	37.7- 72.7	417.3 ef	303.0- 574.7	0.00 a	–	1.17 a	0.4- 3.0	0.00 a	–
May	150.2 cd	115.0- 196.0	41.5 c	29.77- 57.9	31.8 bc	22.4- 45.3	176.5 d	127.9- 244.8	0.50 a	0.10- 3.0	1.33 a	0.49- 4.0	1.67 a	0.68- 4.0
June	105.33 c	80.1- 138.5	33.7 bc	23.88- 47.5	26.8 b	18.6- 38.6	17.0 bc	11.1- 25.9	1.50 a	0.58- 4.0	1.00 a	0.31 3.0	1.67 a	0.68 4.0
July	42.2 ab	31.0- 57.3	16.5 a	11.06- 24.6	3.7 a	1.9- 7.2	0.5 a	0.09- 2.7	1.50 a	0.58- 4.0	0.50 a	0.10- 3.0	0.33 a	0.04- 3.0
August	51.0 b	37.9- 68.7	13.3 a	8.73- 20.4	25.0 b	17.3- 36.2	10.5 b	6.5- 16.9	1.50 a	0.58- 4.0	0.83 a	0.23- 3.0	0.83 a	0.23 3.0
September	42.5 ab	31.3- 57.7	16.8 a	11.30- 25.1	55.3 c	39.9- 76.7	2.3 a	1.0- 5.3	2.00 a	0.88- 5.0	0.17 a	0.01- 3.0	1.17 a	0.40- 3.0
October	198.3 de	152.6- 257.8	21.2 ab	14.52- 30.9	25.8 b	17.9- 37.3	293.7 de	212.8- 405.3	0.83 a	0.23- 3.0	1.67 a	0.68- 4.0	1.17 a	0.40- 3.0
November	289.3 e	223.5- 374.6	73.7 d	54.03- 100.4	5.2 a	2.9- 9.3	538.8 f	391.7- 741.2	0.17 a	0.01- 3.0	0.00 a	–	1.33 a	0.49- 4.0
December	100.67 c	76.5- 132.5	21.5 ab	14.77- 31.3	540.2 de	404.0- 722.2	607.0 f	441.5- 834.6	0.33 a	0.04- 3.0	0.00 a	–	1.00 a	0.31- 3.0

***Footnote***; values are estimated marginal means froma negative-binomial model with log link; CI: confidence interval; confident level used: 0.95; confidence level adjustment with “sidak method” for 12 estimates; significance level used, alpha = 0.05; Means followed by different lowercase letters within a column differ significantly. Means with the similar lowercase letter within a column do not differ significantly

### 3.6. Seasonal abundance of insect pests

The abundance of green leafhopper, WBPH, YSB, RLF, CW, RB, LHC and WLH were found significantly maximum in Aman season, followed by Aus and Boro season (Table 7; Fig 2). The abundance of BPH was found maximum during Aman season. The highest abundance of field cricket was found in Aus season, which was followed by Aman and Boro season, respectively. The abundance of zigzag leafhopper was observed maximum in Aman and Boro season, followed by Aus season (Table 8; Fig 2). Furthermore, it has been demonstrated that the transitional period from Boro to Aus was most favorable for BPH, WBPH, GLH, RB, FC, LHC, YSB and ZzLH (Fig 2). Moreover, the transitional period from Aus to Aman was congenial for CW, WLH and RLF (Fig 2).

**Table 7:** Abundance of insect pests according to seasons.

Season	Green leafhopper		Brown planthopper		White-backed planthopper		Yellow stem borer		Rice leaf folder	
	Estimated	95%CI	Estimated	95%CI	Estimated	95%CI	Estimated	95%CI	Estimated	95%CI
Boro	185.25 a	146- 235	878.19ab	685- 1127	249.67a	183- 342	167.47a	136- 207	19.69 a	15.2- 25.5
Aus	268.57 b	213- 339	829.87a	647- 1065	258.67a	189- 354	162.47a	132- 201	16.13 a	12.2- 21.3
Aman	607.30 c	483- 764	1236.53b	965- 1585	419.20b	307- 572	243.30b	198- 299	35.70 b	28.9- 44.1

**Table 8:** Abundance of insect pests according to seasons.

Season	Caseworm		Rice bug		Long horned cricket		Field cricket		White leafhopper		Zigzag leafhopper	
	Estimated	95%CI	Estimated	95%CI	Estimated	95%CI	Estimated	95%CI	Estimated	95%CI	Estimated	95%CI
Boro	20.86 a	15.7- 27.8	36.44 a	28.4-46.8	10.39 a	7.07- 15.3	22.67 a	18.3-28.1	85.31 a	68.6- 106	106.94ab	88.1- 130
Aus	18.83 a	14.0- 25.3	54.87 b	43.5-69.1	13.67 a	9.58-19.5	58.20 c	50.4-67.2	127.80b	103.6- 158	98.33 a	81.3- 120
Aman	45.10 b	35.5- 57.4	96.80 c	78.1-119.9	54.80 b	41.73-72.0	41.56 b	35.3-49.0	335.60c	275.2- 409	137.36b	113.7- 166

***Footnote***; values are estimated marginal means from a negative-binomial model with log link; CI: confidence interval; confident level used: 0.95; confidence level adjustment with “sidak method” for 12 estimates; significance level used, alpha = 0.05; Means followed by different lowercase letters within a column differ significantly. Means with the similar lowercase letter within a column do not differ significantly

**Fig 2:**
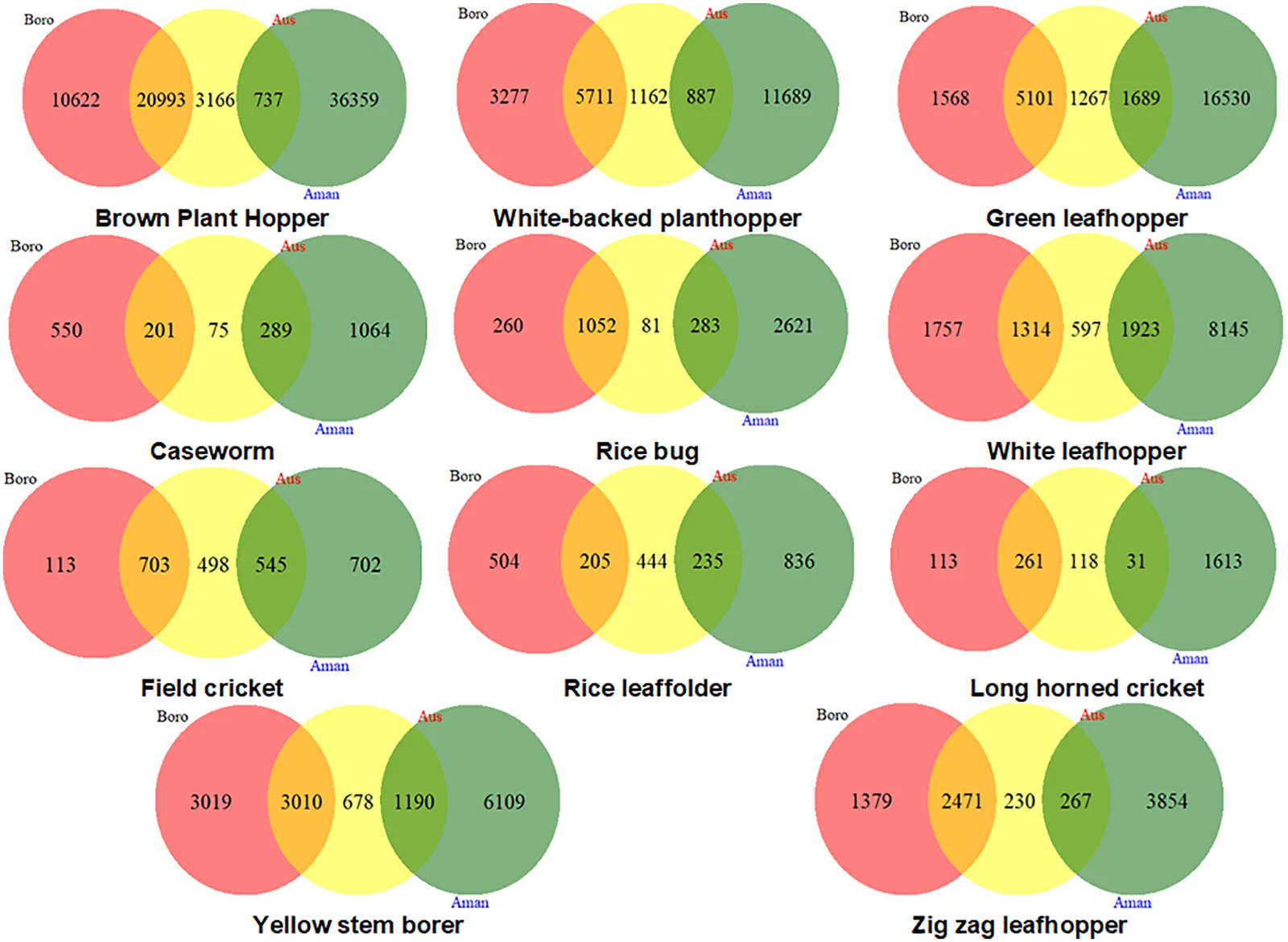
Ven diagram showed the shared and unique insect pests taxa recorded during the irrigated season (Boro), the pre-monsoon season (Aus), the monsoon season (Aman), and the transition period.

### 3.7. Seasonal abundance of natural enemies

The abundance of carabid beetle was found statistically significant and highest in Aman season, followed by Aus and Boro seasons (Table 9; Fig 3). The abundance of ladybird beetle was found maximum in Aman season. But, Boro was the season of significantly maximum StPB abundance, followed by Aman and Aus seasons. The green mirid bug found statistically significant and highest in Boro and Aman season. The abundance of damselfly, dragonfly and spider was found non-significant difference among seasons. Furthermore, the transitional period from Boro to Aus was congenial for CBB, GMB, dragonfly, LBB, StPB and spider. Moreover, the transitional period from Aus to Aman was observed congenial for damselfly (Fig 3).

**Table 9:** Abundance of natural enemies according to seasons.

Season	Carabid beetle		Ladybird beetle		Staphylinid beetle		Green mirid bug		Damselfly		Dragonfly		Spider	
	Estimated	95%CI	Estimated	95%CI	Estimated	95%CI	Estimated	95%CI	Estimated	95%CI	Estimated	95%CI	Estimated	95%CI
Boro	66.53 a	57.1- 77.5	20.08 a	16.2- 25.0	412.61 b	355.5-478.9	205.94 b	165.6- 256	0.36 a	0.7- 1.83	0.44 a	0.10- 1.92	1.06 a	0.41-2.73
Aus	78.93 a	68.12- 91.3	24.07 ab	19.7- 29.4	27.93 a	22.1-35.2	124.37 a	99.3- 156	1.00 a	0.38- 2.65	0.97 a	0.36- 2.61	0.90 a	0.32-2.51
Aman	124.67b	109.5- 141.9	28.30 b	23.6- 34.0	23.00 a	18-29.5	169.17 ab	135.7- 211	1.20 a	0.49- 2.92	0.63 a	0.19- 2.16	0.97 a	0.36-2.61

***Footnote***; CBB: carabid beetle; LBB: ladybird beetle; StPB: staphylinid beetle; GMB: green mirid bug; values are estimated marginal means froma negative-binomial model with log link; CI: confidence interval; confident level used: 0.95; confidence level adjustment with “sidak method” for 12 estimates; significance level used, alpha = 0.05; Means followed by different lowercase letters within a column differ significantly. Means with the similar lowercase letter within a column do not differ significantly.

**Fig 3:**
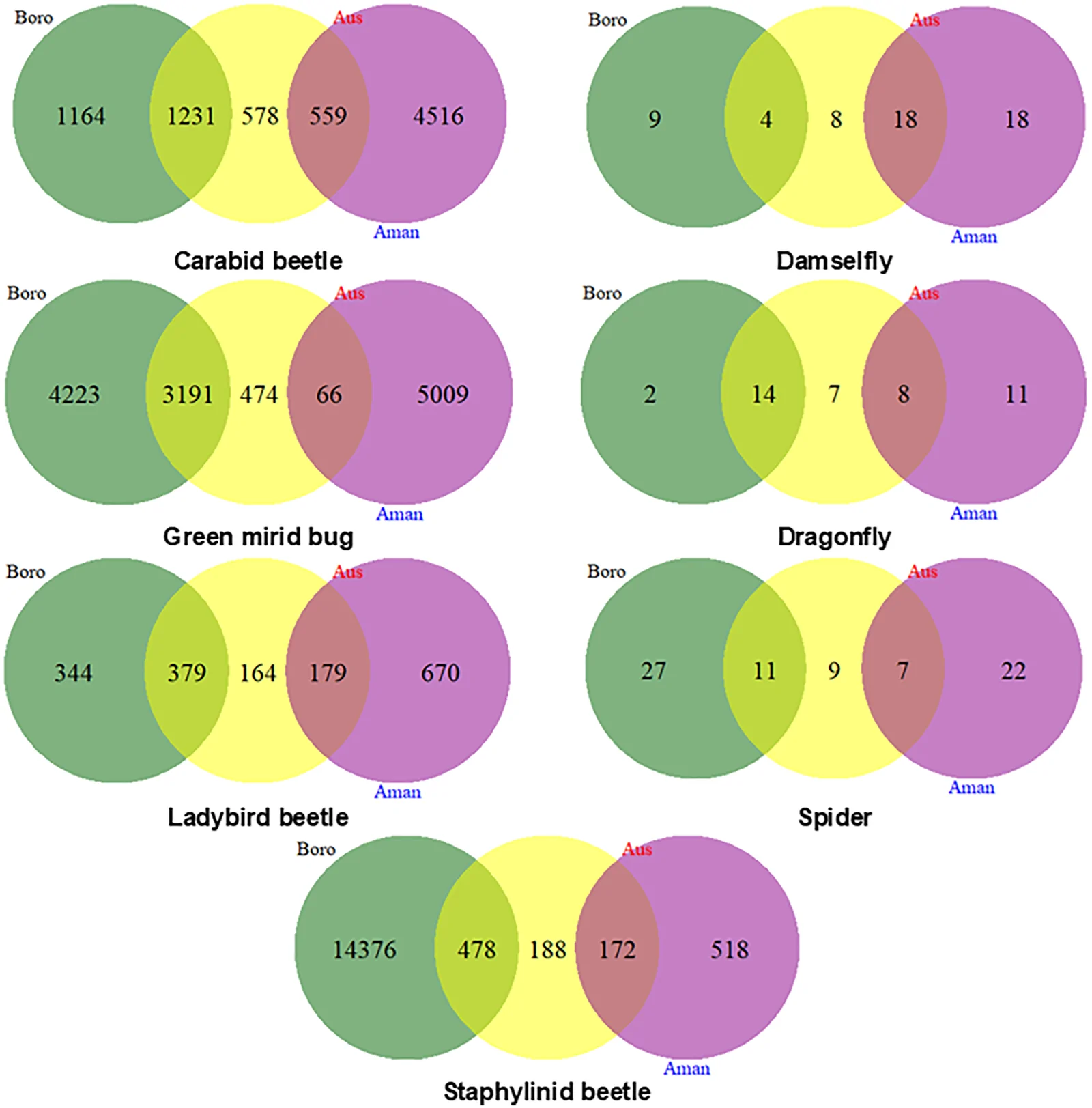
Ven diagram showed the shared and unique natural enemy taxa recorded during the irrigated season (Boro), the pre-monsoon season (Aus), the monsoon season (Aman), and the transition period.

### 3.8. Influence of weather variables on the interactions between insect pests and natural enemies

The abundance of studied insect pests and their natural enemies manifested significant variation with different weather variables (Fig 4). The abundance of dragonfly positive and significantly associated by maximum, minimum and mean temperature. Filed cricket significantly correlated by minimum temperature with positively. The abundance of staphylinid beetle was significantly and negatively associated by maximum, minimum and mean temperature. Moreover, most of the insect pests and natural enemies positively but non-significantly correlated by temperature variables, while green mirid bug and spider have non-significant and negative association with temporal variables. Similarly, rainfall non-significantly and positively associated with the abundance of CW, RLF, WBPH, WLH and GLH while negatively correlated with dragonfly, damselfly, spider, StPB, LBB, CBB, RB, FC, LHC, BPH and ZzLH (Fig 4). Relative humidity significantly and positively correlated with the abundance of damselfly, and negatively with GMB, StPB, BPH and ZzLH. The presence of all insect pests significantly and positively interrelated with each other, except RLF with ZzLH, BPH and WBPH; CW with BPH, and WBPH; LHC with ZzLH. Among the natural enemies the abundance of dragonfly was positively and significantly correlated with the presence of RB, non-significant but positive with others except FC, LHC, CW and RLF. The presence of spider was significantly and positively associated with RLF, CW and LHC; non-significant but positive with FC and RB; and negative correlation showed with other insect pests. Damselfly abundance was positively and significantly correlated with RLF and CW; non-significant but positive with LHC and negative with other studied insect pests. The abundance of GMB was positively and significantly associated with RB, YSB, WBPH, BPH, ZzLH and WLH; non-significantly with GLH and negatively with rest of the studied insect pests (Fig 4). Staphylinid beetle showed non-significant and positive association with YSB, WBPH and BPH; while negative association with RB, FC, LHC, CW, RLF, ZzLH and GLH. The abundance of LBB was positively correlated with the abundance of CW and LHC; non-significant with GLH and negative with other studied insect pests. The abundance of CBB was significantly and positively correlated with RB, FC, YSB, WBPH, BPH, ZzLH, WLH and GLH; and non-significant and negative with RLF, CW and LHC (Fig 4).

**Fig 4:**
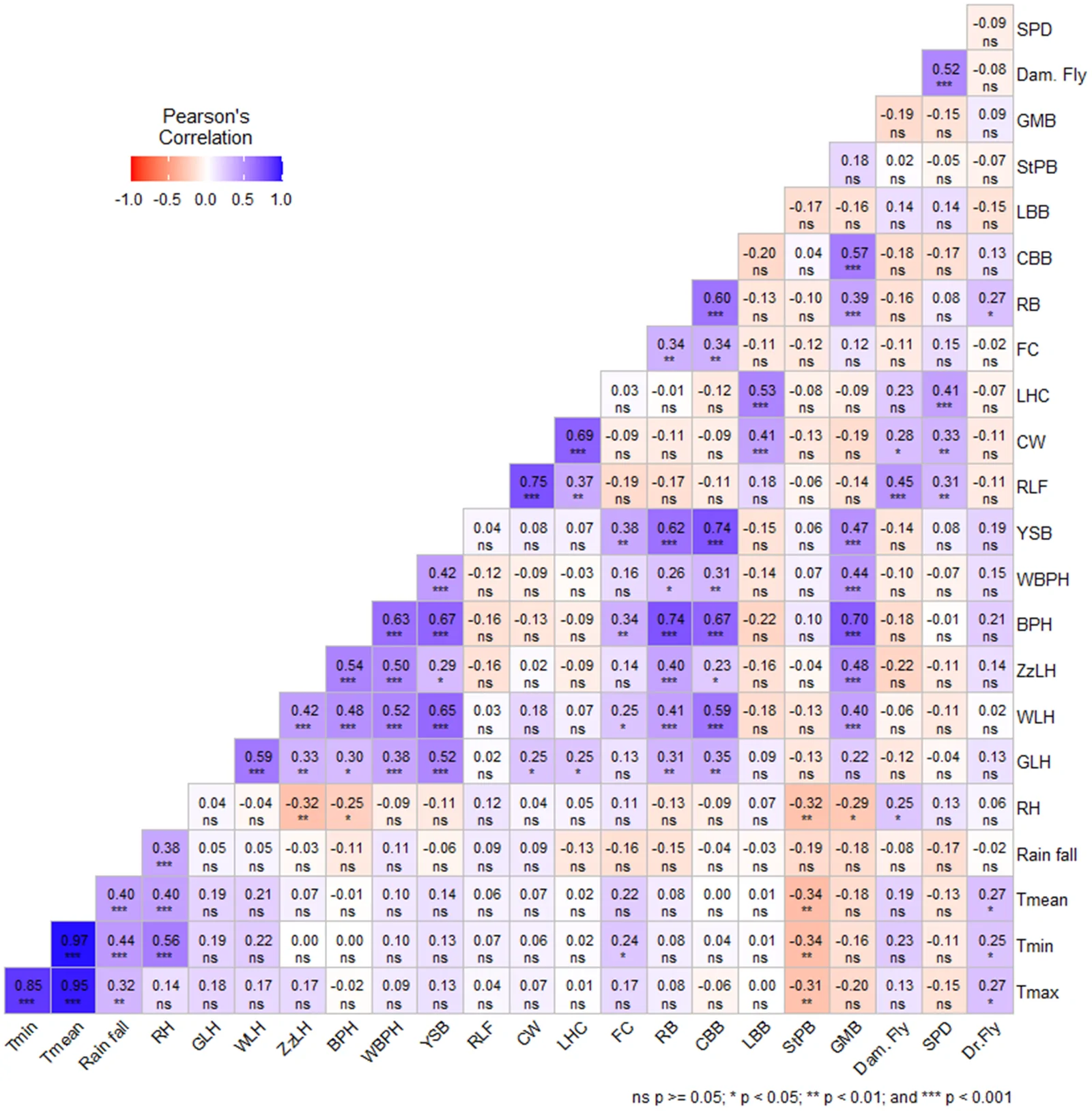
Pearson correlation coefficient of rice insect pest and natural enemies with different weather parameters. ***Footnote***; GLH: green leafhopper; BPH: brown planthopper; WBPH: white-backed planthopper; YSB: yellow stem borer; RLF: rice leaf folder; CW: caseworm; RB: rice bug; LHC: long horned cricket; FC: field cricket; WLH: white leafhopper; ZzLH: zigzag leafhopper; CBB: carabid beetle; LBB: ladybird beetle; StPB: staphylinid beetle; GMB: green mirid bug; Damp. Fly: damselfly; Drag. Fly: Dragonfly; Tmax: maximum temperature; Tmin: minimum temperature; Tmean: mean temperature; RH: relative humidity; ***: significant at 0.1%; **: significant at 1%; *: significant at 5%; ns: non-significant.

## 4. Discussion

### 4.1. Monthly and seasonal dynamics of weather variables

The pattern of observed monthly and seasonal weather variables are consistent with the tropical monsoon climate governing rice based agroecosystems in Bangladesh and similar South Asian regions. Higher maximum, minimum and mean temperatures from March to October, with peaked during from April to July, coincide with the pre-monsoon and monsoon periods. Moreover, the lower temperatures from November to February reflect the irrigated winter season. January is the coldest month in Bangladesh. After winter periods the temperature became increased from March-April and the gap between maximum and minimum temperature observed narrowed, which increase the activity of insect pests due to concludes the hibernation and migration of insect pest towards grasses and rice fields for the initial reproduction phase [3]. The concentration of rainfall and high relative humidity between May to October, especially during June and August, fit to the southwest monsoon period. Therefore, during these months the high humidity and frequent rainfall create favorable microclimatic conditions for herbivorous insect pests and simultaneously influence the activity, survival and dispersal of natural enemies [3,8,15]. Seasonal comparisons of weather variables further revealed that the Aus season experienced with the highest temperature and rainfall, followed by Aman and Boro seasons, which reflects distinct agro climatic situations which influence insect abundance across rice growing seasons. Moreover, Boro season also experienced with very cold temperature and increased high temperature also. Such seasonal thermal periods are well documented and play a critical role in regulating the phenology of rice as well as the population dynamics of insects [3,6].

### 4.2. Diversity index of rice insect

Understanding the ecological interactions of rice insect pests and their natural enemies under changing climatic conditions is essential for the development of effective, ecologically based pest surveillance and management techniques. The six year longitudinal study, spanning from January 2019 to December 2024 offers crucial insights into the seasonal and monthly fluctuations of major insect pests and their natural enemies concerning major climatic factors. The Shannon-Wiener diversity index (H´) serve as a reliable measure for assessing the biodiversity of insect pests in a group. The H´ value of 2.16 signifies a modest diversity of rice insect pests and natural enemies, whereas maximum potential diversity (H max = 2.89) and evenness index (E = 0.75). The brown plant hopper (Pi = 0.3469) exhibited significance prevalence among insect pests, representing the largest proportion to overall community diversity. This supremacy highlights that BPH is presently the principal ecological catalyst of pest pressure in study area of Bangladesh, especially in intensive rice farming, elevated nitrogen fertilizer application and conducive climatic conditions that enhance BPH proliferation while compromising ecosystem stability [3,16,17]. The green leafhopper (Pi = 0.1267), white-backed planthopper (Pi = 0.1084) and yellow stem borer (Pi = 0.0679) were the subsequent most prevalent pests. Among the natural enemies, the staphylinid beetle (Pi = 0.0766) was the most abundant, and green mirid bug (Pi = 0.0614) and carabid beetle (Pi = 0.330) were the subsequent natural enemies.

### 4.3. Monthly, seasonal and transitional period abundance of insect pests and natural enemies in relation to climate

The monthly significant variation in the abundance of all insect pests highlights the strong influence of temporal climatic variability (Table 4). During September-November and April-May, the higher abundance of planthoppers and leafhoppers (GLH, BPH, WBPH, WLH and ZzLH) observed in the study period, which corresponds with moderate to high temperature and high humidity periods, conditions commonly known to favor of hopper survival, reproduction and migration [3,6,18]. Peak activity of BPH, GLH and WBPH in October-November closely align with the reproductive and maturity stage of Aman season when plant growth and favorable microclimates enhance host suitability [3]. Similarly, the increased abundance of rice leaf folder and caseworm during September-October reflects their association with weather variable like humidity and dense of crop canopy, during the reproductive stage (Table 1) of Aman rice [3,4,9]. Seasonal analysis revealed that most of the insect pests attained their peaked abundance during the Aman season, followed by Aus and then Boro seasons (Table 10). Aman rice is typically cultivated under rain-fed conditions with higher humidity, temperature and complex canopy structures, which favor colonization and population buildup. Aus rice also cultivated under heavy rain with high temperature and relative humidity. Moreover, the peaked populations of rice bug and long-horned cricket in October-November, coinciding with grain filling and maturity stages of Aman rice and soft rice grain in panicle provide optimal feeding resources [3,11,19]. The peaked abundance of field crickets during warmer month (May-July), highlighting their preference for narrowed minimum temperature with maximum temperature and relatively soil conditions. In contrast, Boro rice cultivated during cooler. Less humidity and relatively drier months under irrigated conditions, which responsible for comparatively lower abundance of harmful pests.

**Table 10:** The peak activity period of key insect pests from January 2019 to December 2024.

Name of the insects	Month	Season	Temperature (°C)			Relative Humidity (%)
			Maximum	Minimum	Mean	
Yellow stem borer	May, October, November, September	Aman	30.93-34.66	20-27.09	25.45-30.25	65.83-78.82
Brown planthopper	November, October, May	Aman, Boro	30.93-34.66	19.97-25	25.45-29.80	65.83-73.54
White-backed planthopper	October	Aman	33.15	24.99	29.07	73.54
Rice leaf folder	September, October	Aman	33-34	25-27	29-30	66-73.54
Rice Bug	October, November, May	Aman> Boro	30.93-34.66	19.97-25	25.45-29.80	65.83-73.54
Green leaf hopper	October>November	Aman	30.93-33.15	19.97-25	25.45-29.07	65.83-73.54
Rice caseworm	October, November	Aman	30.93-33.15	19.97-25	25.45-29.07	65.83-73.54

Moreover, proliferation of insects are significantly influenced by the transitional periods between cropping seasons. The transition period from Boro to Aus was consistently favorable for insects like planthoppers, leafhoppers, rice bug, stem borer and crickets may be due to overlap of residual host plants, increasing humidity and rising temperature which responsible to facilitate survival, migration and rapid population growth [3,11,19]. In addition, the transition between Aus-Aman favored rice leaf folder, rice caseworm and white leafhopper.

Furthermore, natural enemy populations also exhibited strong temporal and seasonal pattern. The abundance of natural enemy closely related with the abundance of harmful species. Predators like carabid beetles and ladybird beetles peaked mainly in October-November, coinciding with the peaked abundance of prey insects during the Aman season. This temporal composition suggests density dependent responses of predators to prey availability a key composing mechanism in rice agroecosystems [20,21]. The maximum abundance of staphylinid beetles exhibited during December-March month (winter season) which is under the Boro season, indicating their preference for lower temperatures which further confirmed by their negative association with temperature variables. Moreover, odonates (dragonflies and damselflies) were most abundant during the Aus and the Aman seasons, coinciding with high humidity, rainfall and the availability of aquatic habitats made by monsoon rainfall, which are essential for their development of larva [3,22,23]. Spider, non-significant monthly and seasonal variation, maintained relatively stable populations throughout the year, which indicating their normal predatory behavior and resilience across varying climatic conditions. Spider an ecological indicator, which reflects environmental health and biodiversity due to ability to thrive across seasons underscores their role in pest management and balance of the ecosystem [3,12,24].

Weather variables poses both direct and indirect effects on insect pests as well as natural enemies abundance. Temperature positively associated with dragonflies and field cricket abundance, highlighting temperature dependent development and flight activity of odonates [22]. Moreover, temporal variable were non-significantly but positively associated with most of the insect pests and natural enemies, except staphylinid beetle, green mirid bug and spider. In contrast, the significantly negative correlation of staphylinid beetles with temporal variables suggesting their vulnerability of thermal stress. However, rainfall exhibited predominantly non-significant association with insect pests and natural enemies but tended to favor foliage-feeding insects like rice leaf folder and caseworm, likely through enhanced host plant growth and succulent leaves. Furthermore, rainfall negatively correlated numerous predator, due to disturbance physically, reduced efficiency of foraging or mortality of larvae [25]. On the other-hand, relative humidity a key climatic driver, positively influencing the abundance of damselfly while negatively affecting BPH, ZzLH, YSB and RB and predators like CBB, StPB and GMB.

Our findings showed significant positive interrelationships among most insect pests, highlight shared responses to favorable environmental conditions as well as crop phenology rather than direct boosting [3,11]. In contrast, natural enemies exhibited selective associations with specific insects likes; spiders were positively correlated with leaf folder, caseworm, yellow stem borer, rice bug and long-horned cricket highlight the vast key role of spiders for inhibition of pest populations. Carabid beetle and green mirid bugs were showed positive association with planthoppers and leafhopper, which consistent with their role as key predator of sap-feeding insects and mostly prey egg, larva and adults of these insects [8,25]. Damselflies exhibited positive correlation with rice leaf folders, caseworm and long horned cricket; while dragonflies exhibited positive association, rice bug, stem borer, planthoppers and leafhoppers. Conversely, natural enemy showed negative correlations also with insect pests. These intricate correlation among natural enemies with multiple pest species indicate their broad predatory range.

### 4.4. Limitations of present study

Despite present study provides a comprehensive assessment of insect pests and natural enemy dynamics for six years by using light trap, but it was performed at a single monitoring site utilizing a solitary light trap. Moreover, dependence on light trap data could lead to an underrepresentation of non-phototactic or day active species and may not accurately reflect true field numbers. Consequently, the observed abundance trends and climatic associations mostly represent the ecological circumstances of the study location and should not be generalized to all rice cultivation areas. In addition, insect populations are affected by local agro-ecological factors such as planting time, cropping intensity, rice cultivars, adjacent flora, landscape diversity, management techniques and regional climate fluctuations. Moreover, agricultural performance metrics like grain production, yield reduction, and economic thresholds were not explicitly assessed in conjunction with insect populations, complicating the quantification of the exact productivity effects of certain pest infestations. Although the extensive temporal dataset enhances assurance in the seasonal patterns observed at this site, the quantitative estimates of insect population may differ under different environmental circumstances. Future research should incorporate direct field sampling techniques, assessment of yields and economic injury threshold analysis to enhance pest prediction models.

### 4.5. Implication of light trap for integrated pest management

Light trap is an effective instrument for monitoring of insect population in a particular crop field. Particularly, nocturnal insects including planthopper, leafhoppers, rice leaf folder, caseworm, stem borer are capture by light trap, and can also use as a pest management tools in IPM strategy when the activity of insect pests are peaked [26]. In IPM strategy, the proper monitoring of insect pest populations plays a crucial role and light trap is one of the appropriate monitoring tools [27,28]. We used light trap to track the monthly and seasonal abundance of rice insect peat and natural enemies. Light trap captures mainly nocturnal species, that’s the main drawback of light trap. Moreover, the efficacy of light trap influence by night length, trap design, size, height, kind of bulbs use and weather variables including temperature, humidity, rainfall during trapping, wind, cloud cover and moonlight. The synchronization between pests and natural enemies highlights the potential of ecologically based IPM strategies which leverage climatic forecasting and enhance sustainable rice production under changing climate scenario.

## 5. Conclusion

This six year light trap monitoring study at Bangladesh Rice Research Institute (BRRI), Gazipur demonstrated that seasonal dynamics of insect pests and natural enemies under the local rice agro-ecosystems. The peak activity period of insect pests found in two times in a year; a) April-May and b) September-November and coinciding with the reproductive stage of Boro and Aman season, respectively. Among the seasons, Aman season found as most abundance season of insect pests. The observed temporal patterns also showed clear associations with seasonal weather conditions, suggesting that climatic variability and crop phenology jointly influence insect activity at BRRI Gazipur monitoring site. So, the identification of peak period time including months and seasons particularly the Aman season and the transitional periods, provides critical windows for proper monitoring and intervention of insects. So, by integrating proper monitoring, conservation of natural enemy, long-term climatic scenario and IPM strategy may reduce pest populations naturally. Future studies integrating light trap monitoring with field infestation assessments, economic threshold analyses and evaluations of biological control and IPM interventions will be essential to determine whether these seasonal patterns can be translated into practical pest management recommendations.
