# Supplementary material for: Influence of weather variables on population dynamics of rice insect pests and their natural enemies: A six year light trap study in Gazipur, Bangladesh
Source: PLoS One. 2026 Oct 7;21(10):e0358423. doi: 10.1371/journal.pone.0358423 (PMC13645023; doi:10.1371/journal.pone.0358423)
Supplement: Supplementary file 1 [file pone.0358423.s002.docx]

**Editor in Chief,**

Thank you for your consideration and nice observation of our present manuscript. The author’s response according to the reviewer comments are present below, the correction (RED color) also made in the word file manuscript.

**Reviewer comments and Author response**

**Major comments**

**1. Study design and scope**

Only one light trap was used at one location. Repeated observations through time do not provide spatial replication. The title, abstract, discussion, and conclusion should clearly state that this is a single-site light-trap study in Gazipur. Broad statements about rice agroecosystems, Bangladesh, or tropical rice systems should be reduced.

**Author Response**: Thank you for your suggestion. We have revised the manuscript throughout to clearly define the scope of inference according to your suggestion. The study area has been explicitly described as a six year, single site light trap monitoring. We have changed the title also.

**2. Sampling effort is not clear**

The authors state that insects were collected daily, but they do not report the total number of operational trap nights, missing nights, power failures, bulb failures, maintenance periods, or other interruptions. The light operated for 10-12 hours, which means sampling effort was not always equal. The authors must report the number of trapping nights and hours for every sampling period and standardize the catches by effort or include effort as an offset in the model.

**Author Response**: Thank you for your valuable observation. We agree that a clear description of sampling effort is essential for interpreting long term light trap data. We have substantially revised these information including “light trap operation hour”, total trapping nights in our revised manuscript

**3. Explain exactly how the data were summarized**

It is not clear what the values in Tables 3-6 represent. They may be monthly totals, means per night, means of six yearly totals, or another measure. The authors must define the basic observation unit, show how daily catches were converted into monthly and seasonal values, report sample sizes, and give measures of variation such as standard error or confidence intervals.

**Author Response**: Thank you for your nice observations. In the revised manuscript, we now explicitly define the basic observational unit as the daily light trap catch (Number of individuals per operational trap night). Firstly, daily insect counts were recorded for each operational trap night then aggregated to obtain monthly mean catches per trap night. Monthly values were subsequently grouped according to the rice growing seasons. Standard error and co-efficient of variation have also been added to the revised tables to improve the tables.

**4. The statistical analysis must be replaced**

The response variables are insect counts. Count data are commonly non-normal, overdispersed, and sometimes zero-inflated. In addition, observations from consecutive days or months are not independent. A simple ANOVA with LSD comparisons and Pearson correlations is not sufficient. The authors should use a negative-binomial generalized linear mixed model or a generalized additive mixed model. The model should account for year, seasonality, unequal trapping effort, and temporal autocorrelation. Model assumptions and diagnostics must be reported.

**Author Response**: Thank you for this valuable comments. We have revised the statistical analysis for the monthly abundance of insect pests that explicitly account for over-dispersion. The daily trap night catch was the fundamental observational unit. For each insect taxon, abundance was analysed using a negative binomial generalized linear model (GLM). Details was included in our revised manuscript.

**5. Monthly and seasonal tests are not described**

The Methods section does not explain the tests that produced the P values, LSD values, and letter groupings in Tables 3-6. The authors must state the exact statistical test, model formula, post hoc method, software packages, sample size, and adjustment for multiple testing. Fisher's LSD across many months and many taxa can strongly increase false-positive results and should not be used without proper control.

**Author Response**: For monthly abundance we used negative binomial GLM in our revised manuscript. So, we have changed the table from Table 4 to Table 7 in our revised manuscript (Table 3 was newly added, that’s why table number has been changed). Thank you for your suggestions.

**6. The rice seasons overlap**

Table 1 shows that Boro, Aus, and Aman overlap in several months. Therefore, the same insect observations may be included in more than one season. The Venn diagrams do not solve this problem and do not provide independent seasonal comparisons. The authors should use actual planting, crop-stage, and harvest records for each year and define non-overlapping analytical periods. If these records are unavailable, month or date should be analysed directly, and the general rice calendar should be used only for interpretation.

**Author Response**: Thank you for your suggestion. We agree that the overlapping cultivation periods of Boro, Aus, and Aman rice in Bangladesh can result in temporal overlap in insect occurrence and that this issue needs to be distinguished from independent statistical comparisons among seasons. We have therefore revised the manuscript to clarify the analytical role of season, month, and the Venn diagrams. In the revised manuscript, seasonal abundance is presented separately according to the actual rice-growing calendar followed at the study site, and the Bangladesh rice calendar is used primarily to facilitate ecological interpretation of insect occurrence in relation to rice production periods. We acknowledge that the general rice calendar cannot, by itself, establish completely independent temporal periods when different rice crops are simultaneously present in the landscape. We used Venn diagram only for comparison of overlapping seasons and to show how season plays role for the transmission of insects transmitted from one season to another season. Because rice cultivation in Bangladesh involves overlapping cropping calendars, many insect species occur across multiple crop seasons. The Venn diagrams were specifically designed to visualize these ecological relationships rather than temporal abundance. Importantly, the Venn diagrams are not used as statistical tests of independent seasonal differences or as evidence of a causal “season effect.” Rather, they are retained as a complementary descriptive visualization to illustrate the shared and unique occurrence of insect taxa recorded during the Boro, Aus, and Aman production periods. Thus, the Venn diagrams address community overlap in taxonomic occurrence and should not be interpreted as resolving temporal pseudo-replication arising from overlapping rice seasons.

**7. Weather effects are confounded with season**

Temperature, rainfall, humidity, month, and rice season are closely related. The three temperature variables are also strongly correlated with each other. The authors should not use maximum, minimum, and mean temperature together without evaluating collinearity. They should choose biologically meaningful predictors, report VIF or another collinearity assessment, separate seasonal patterns from additional weather effects, and examine reasonable lagged effects of weather on insects.

**Author Response**: Thank you for your important suggestion. We would like to clarify, however, that the primary purpose of presenting the weather data in this manuscript was descriptive rather than predictive or causal. Specifically, the weather variables were summarized as monthly average meteorological conditions to provide environmental context for the observed seasonal and monthly patterns of rice insect abundance. They were not intended to constitute a multivariable predictive model in which maximum temperature, minimum temperature, mean temperature, rainfall, relative humidity, month, and season were simultaneously interpreted as independent explanatory variables.We acknowledge that a formal VIF/collinearity assessment and lagged modelling would be appropriate if the objective were to quantify the independent contribution of individual weather variables to insect abundance. However, such an analysis was not part of the original study design, and the available weather dataset was summarized at the monthly level

**8. Correlation is over-interpreted**

A positive correlation between a pest and a predator does not prove a predator-prey relationship. Both groups may simply respond to the same month, weather, crop stage, or trapping conditions. A negative correlation does not prove pest suppression. The manuscript should replace words such as 'influence', 'regulate', 'suppression', 'dependency', and 'biological control' with 'association' unless stronger analysis supports a causal statement. Predator-prey relationships should be treated as exploratory hypotheses.

**Author Response**: Thank you for your suggestions. You are alright. We have replaced the mention words from our revised manuscript. Also, improved correlation related section in both results and discussion where necessary.

**9. Light-trap catch is not the same as field population density**

Light traps measure the activity and attraction of phototactic insects. Catch can change because of night length, rainfall during trapping, wind, cloud cover, moonlight, bulb condition, surrounding lights, and insect flight activity. The authors should use terms such as 'light-trap catch' or 'activity-density' rather than claiming that the trap directly measured the true field population. The statement that a light trap captures about 94% of the total field population is very doubtful and should be verified carefully or removed.

**Author Response**: Thank you for your suggestion. We have removed the statements that a light trap captures about 94% of the total field population from the section 4.3. Implication of light trap for integrated pest management.

**10. Crop and management information is missing**

The paper links peaks to crop reproductive stages, but it does not provide actual yearly data on rice variety, planting date, crop stage, area planted around the trap, irrigation, insecticide application, fertilizer, harvest, fallow periods, or changes in nearby land use. These variables may strongly affect catches. The authors should provide these records. Without them, conclusions about crop stage and transitional periods must be weakened.

**Author Response**: Thank you for your suggestions. We have provided the rice season data in table 1. Although, rice variety, actual planting date, irrigation, fertilizer and insecticide data were missing, we have added some point regarding these in our revised manuscript in materials and methods section.

**11. Taxonomic identification needs much more detail**

Most taxa are presented only by common names or broad groups. The authors should provide scientific names where possible, identification keys, the names or qualifications of identifiers, treatment of damaged or uncertain specimens, and voucher deposition information. Broad groups such as spiders, carabids, staphylinids, and ladybird beetles include species with different ecological roles. Also, spiders are not insects; use 'arthropod natural enemies' instead of 'beneficial insects'.

**Author Response**: Thank you for your insightful comments. We have now provided scientific name with identifiers in our revised manuscript in Table 3. We have also used the term “arthropod natural enemy’ where applicable.

**12. Figures and tables should be redesigned**

Figure 2 should show months in calendar order from January to December. Figure 3 uses violin plots with very few data points and allows rainfall density below zero, which is impossible. Figures 4 and 5 use Venn diagrams for seasonal totals, but these figures hide time, effort, and uncertainty. Replace them with chronological time-series plots and model-based seasonal estimates with 95% confidence intervals. Every table should report units, n, variation, and the statistical model used.

**Author Response**: Thank you for your nice and insightful observations. There are six climatic seasons in Bangladesh, summer (from mid April to mid June), rainy (from mid June to mid August), autumn (from mid August to mid October), late autumn (from mid October to mid December), winter (from mid December to mid February) and spring (from mid February to mid April). These six seasons represent ecologically distinct climatic periods that strongly influence rice phenology, insect development, migration and natural enemy activity in Bangladesh. Therefore, organizing the data according to the national climatic seasons provide greater ecological relevance than chronological arrangement. We have changed the Figure 3 (for rainfall).

The purpose of Figure 4 and Figure 5 is not to describe temporal population dynamics but rather to summarize species occurrence and overlap among rice growing seasons (Aus, Aman and Boro). Unlike time series analyses, Venn diagrams provide an intuitive visualization of shared insect species among rice seasons; season specific species richness and ecological overlap of pest and beneficial arthropod communities. Because rice cultivation in Bangladesh involves overlapping cropping calendars, many insect species occur across multiple crop seasons. The Venn diagrams were specifically designed to visualize these ecological relationships rather than temporal abundance. Hopefully you considered.

**13. The IPM conclusions are too strong**

The study did not measure field infestation, crop damage, yield loss, economic thresholds, predator feeding, or the effect of an intervention. Therefore, it cannot demonstrate that natural enemies controlled pests or that insecticide use can be reduced. The results may help identify periods for closer local monitoring, but management recommendations must be presented as hypotheses that require field validation.

**Author Response**: Thank you for your comment. According to your suggestion, we have restructured our conclusion section.

**14. Data availability is inadequate**

The statement that all data are presented in the manuscript is not correct because only aggregated results are shown. The authors should deposit the minimum date-level dataset, weather data, sampling-effort information, taxon dictionary, and analysis code in a public repository and provide a permanent link or DOI. 'Data available on request' is not sufficient for reproducibility.

**Author Response**: The insect counts data, meteorological data used in this study and analytical code (R scripts) supporting the statistical analyses are not currently available in a public repository. However, all datasets and analytical code are available from the corresponding author upon reasonable request. This ststements has been added to the manuscript unser the **data availability** section.

**15. Field permission and ethics information**

The submission lists the ethics statement as not applicable, but the study involved field collection of insects. The authors should state that permission was obtained to operate the trap and collect insects at the BRRI site, name the responsible institution, and provide a permit number if one was required. If no permit was required, this should be stated clearly.

**Author Response**: Thank you for your comments. The authored are working under Bangladesh Rice Research Institute (BRRI). And the research field is belongs to BRRI, and this research was a part of regular monitoring of rice insect pest. So, no permission is required for light trap setting and insect collections.

**16. English and scientific writing**

The manuscript contains many grammatical errors and unclear sentences in the abstract, introduction, methods, results, discussion, and conclusion. Terminology is also inconsistent. The paper requires complete English-language editing after the analysis is corrected. Use 'insect pests' instead of 'harmful insects', 'abundance' or 'catch' instead of 'prevalence', and 'rainfall' instead of 'rain fall'.

**Author Response**: Thank you for your insightful suggestions. We have improved grammar and sentence throughout the revised manuscript. We also used suggested terminology.

**Recommended analysis**

·       Use trap-night as the preferred observation unit. If monthly data are used, retain the number of operational nights.

**Author Response**: Thank you for your suggestion. We basic observational unit of our collected data was daily trap night catch. Daily insect counts were averaged to obtain monthly mean catches ‘trap night^-1^”. Furthermore, monthly values were subsequently grouped according to the three rice growing seasons (Boro, Aus and Aman) for seasonal analyses. These information was added in our revised manuscript.

   Analyse each taxon with a negative-binomial GLMM or GAMM. Test whether a zero-inflated model is needed.

**Author Response**: Thank you for your suggestion. We have used GLM and also performed a zero inflated model where necessary.

·       Include an offset for trapping hours or operational nights.

**Author Response**: Thank you for your suggestion. We have added trapping hours

·       Represent seasonality with month, a cyclic smooth, or clearly defined non-overlapping periods.

**Author Response**: Thank you for your valuable suggestion. In Bangladesh, rice is cultivated in three agronomic seasons; Boro (November-May); Aus (April-August) and Aman (July-November). These cropping seasons are defined according to the national rice production calendar and are widely adopted in agronomic and entomological studies. However, unlike six distinct climatic seasons in Bangladesh, the rice growing seasons overlap temporally, particularly Aus with Boro (April-May) and again Aus with Aman (July-August) (Table 1). Consequently, assigning each month exclusively to a single rice growing season would not accurately represent the agricultural production system or the ecological conditions experienced by rice insect pests. To address this issue, we retained the standard rice growing seasons because they are biologically meaningful and directly correspond to crop phenology and pest occurrence. To explicitly account for the overlapping cultivation periods, we incorporated a triple Venn diagram to illustrate the shared and unique occurrence of insect pests and natural enemies among the three rice growing seasons. This graphical approach complements the monthly analyses by visualizing species associations across overlapping cropping periods rather than forcing months into mutually exclusive seasonal categories.

**Minor and editorial comments**

1.     Revise the title to identify the six-year, single-site light-trap design.

**Author Response**: Thank you for your suggestion. We have revised the title which represent six year data collected from single location.

2.     The abstract should state the study site, sampling method, observation unit, model used, and key numerical results.

**Author Response**: Thank you for your suggestions. The sampling method was light trap catch night^-1^ during the study periods at Bangladesh Rice Research Institute (BRRI), Gazipur. We also added numerical results in our revised abstract.

3.     The introduction should end with clear hypotheses or predictions rather than only general objectives.

**Author Response**: Thank you for your suggestion. We have added hypothesis in the introduction section.

4.     Line 55 contains an incomplete expression: 'yield losses year-1'.

**Author Response**: Thank you for your observation. We have made the sentence complete and meaningful.

5.     Describe the weather station, instruments, recording frequency, data quality control, and missing weather values.

**Author Response**: Weather data were collected from the “Davis automatic weather station” operated by Entomology division located at the research field of Bangladesh Rice Research Institute (BRRI), Gazipur. The station continuously recorded daily maximum, minimum and mean temperature, rainfall, relative humidity, sunshine duration using calibrated meteorological sensors. From which daily observations were recorded data every one hour. Daily observations were aggregated into monthly means (cumulative monthly rainfall) for analysis. All weather records were checked for completeness and consistency before analysis. Missing values were rare (<1%) and were replaced using another “Davis automated weather station” data and no missing metrological values were included in the final dataset used for analysis.

6.     Check whether 'penicillium light trap' is the correct trap name.

**Author Response**: Thank you for identifying this error. We carefully reviewed the manuscript and confirmed that “penicillium light trap” was an inadvertent typographical error. The term has been corrected to “pennsylvania light trap” throughout the manuscript.

7.     Correct 'net bug' if 'net bag' was intended.

**Author Response**: Yes, its net bag. Thank you.

8.     Give the trap dimensions, bulb manufacturer or type, wavelength or spectral information if available, and replacement schedule.

**Author Response**: A Pennsylvania type light trap was installed at two m above the ground surface. The trap consisted of a metal frame equipped with a 100-W tungsten bulb. The bulb emitted broad spectrum visible light (approximately 400-700 nm). The bulb was replaced whenever its illumination visibly decreased or immediately after failure to maintain uniform trapping efficiency during the study period.

9.     State how specimens were killed, preserved, identified, counted, and stored.

**Author Response**: Thank you for your query. The specimens were kept and stored in a deep freezer (-20°C) after collection for confirming dead. The identification was done as per (Heinrichs, 1994).

10.  State whether very large catches were subsampled and how counting accuracy was checked.

**Author Response**: No subsampled was done for large catches. Counting was confirmed by counting two times.

11.  Report the R version and package versions. RStudio is only the interface, not the statistical software itself.

**Author Response**: Thank you for your observation and nice suggestion. Accordingly, we have revised the statistical analysis section to report the version of the R statistical computing environment together with principal R packages and their versions used for data analysis and visualization.

12.  Use chronological month order in all figures and tables.

**Author Response**: Thank you for your suggestion. In our revised manuscript, we used chronological month for all figures and table.

13.  Use 'damselfly' and 'dragonfly' consistently.

**Author Response**: We have used damselfly and dragonfly consistently in the revised manuscript.

14.  Define every abbreviation in each table or figure legend.

**Author Response**: Thank you for your suggestion. We have defined the abbreviation as footnote for each table and figure.

15.  Check Table 7 carefully because some reported temperature ranges appear inconsistent.

**Author Response**: Thank you for your suggestion. We have checked the table carefully and made corrections where needed. After revision its now Table 8. Thank you.

16.  Correct the author-name inconsistency 'Najmul/Nazmul'.

**Author Response**: Correct spelling “Md Najmul Bari”

17.  Correct 'Principle Scientific Officer' to 'Principal Scientific Officer'.

**Author Response**: We have made correction.

18.  Correct spelling errors in affiliations, including 'University'.

**Author Response**: We have made correction regarding ‘University’.

19.  Check all references, DOI formats, journal names, and whether each reference actually supports the statement cited.

**Author Response**: Thank you for your suggestions. We have checked all these in our revised manuscript and rearrange references section according to journal format.

20.  Shorten the discussion and organize it around findings supported by the revised models.

**Author Response:** Thank you for your suggestion. We have restructured the discussion section supported by revised analyses.

21.  Add a clear limitations paragraph before the management implications.

**Author Response**: Thank you for your nice suggestion. We have added a limitation paragraph before the management implications.

22.  Rewrite the conclusion so that it applies only to the BRRI Gazipur light-trap series.

**Author Response**: Thank you for your suggestion. We have substantially revised the conclusion to ensure that it is explicitly limited to the findings obtained from the six year light trap monitoring series conducted at BRRI Gazipur.

Thank you for your nice cooperation and consideration. Therefore, the corrections are incorporated in the revised manuscript. We believe these revisions address your concerns and improve the manuscript.

Thank you once again for your valuable feedback. We look forward to your feedback.

Sincerely,

The Authors
