# Supplementary material for: Influence of weather variables on population dynamics of rice insect pests and their natural enemies: A six year light trap study in Gazipur, Bangladesh
Source: PLoS One. 2026 Oct 7;21(10):e0358423. doi: 10.1371/journal.pone.0358423 (PMC13645023; doi:10.1371/journal.pone.0358423)
Supplement: Peer review history

# Editor report: Decision Letter 0

**DOI:** 10.1371/journal.pone.0358423.r001

**Authors:** Mostafa Ghafouri Moghaddam

14 Jul 2026

Dear Dr. Roy%,

Thank you for submitting your manuscript to PLOS ONE. After careful consideration, we feel that it has merit but does not fully meet PLOS ONE’s publication criteria as it currently stands. Therefore, we invite you to submit a revised version of the manuscript that addresses the points raised during the review process.

==============================

**Manuscript number: PONE-D-26-19222**

*Influence of weather variables on population dynamics of rice insect pests and their natural enemies in rice agroecosystem*

<h1>**Recommendation** </h1>

**Major revision**

The study has useful potential because it covers six years and includes both rice pests and natural enemies. However, the manuscript cannot be accepted in its present form. The main problems concern the statistical analysis, overlapping rice seasons, unclear sampling effort, overinterpretation of correlations, limited taxonomic information, and incomplete reporting. The paper should be reconsidered only after a full reanalysis and substantial rewriting.

<h1>**General comments** </h1>

This manuscript presents insect catches from one light trap at the Bangladesh Rice Research Institute in Gazipur from January 2019 to December 2024. The topic is relevant to pest monitoring and integrated pest management. The long monitoring period is a strength. Nevertheless, the study uses one trap at one location, and the results therefore describe a local light-trap time series. They should not be presented as general population dynamics for all rice agroecosystems.

The manuscript currently reports descriptive plots, Pearson correlations, monthly comparisons, LSD values, and seasonal Venn diagrams. These methods do not adequately analyse repeated count data collected over six years. The authors should return to the daily or monthly raw data, clearly define the observation unit and sampling effort, and use an appropriate count time-series model.

<h1>**Major comments** </h1><h2>1. Study design and scope</h2>

Only one light trap was used at one location. Repeated observations through time do not provide spatial replication. The title, abstract, discussion, and conclusion should clearly state that this is a single-site light-trap study in Gazipur. Broad statements about rice agroecosystems, Bangladesh, or tropical rice systems should be reduced.

<h2>2. Sampling effort is not clear</h2>

The authors state that insects were collected daily, but they do not report the total number of operational trap nights, missing nights, power failures, bulb failures, maintenance periods, or other interruptions. The light operated for 10-12 hours, which means sampling effort was not always equal. The authors must report the number of trapping nights and hours for every sampling period and standardize the catches by effort or include effort as an offset in the model.

<h2>3. Explain exactly how the data were summarized</h2>

It is not clear what the values in Tables 3-6 represent. They may be monthly totals, means per night, means of six yearly totals, or another measure. The authors must define the basic observation unit, show how daily catches were converted into monthly and seasonal values, report sample sizes, and give measures of variation such as standard error or confidence intervals.

<h2>4. The statistical analysis must be replaced</h2>

The response variables are insect counts. Count data are commonly non-normal, overdispersed, and sometimes zero-inflated. In addition, observations from consecutive days or months are not independent. A simple ANOVA with LSD comparisons and Pearson correlations is not sufficient. The authors should use a negative-binomial generalized linear mixed model or a generalized additive mixed model. The model should account for year, seasonality, unequal trapping effort, and temporal autocorrelation. Model assumptions and diagnostics must be reported.

<h2>5. Monthly and seasonal tests are not described</h2>

The Methods section does not explain the tests that produced the P values, LSD values, and letter groupings in Tables 3-6. The authors must state the exact statistical test, model formula, post hoc method, software packages, sample size, and adjustment for multiple testing. Fisher’s LSD across many months and many taxa can strongly increase false-positive results and should not be used without proper control.

<h2>6. The rice seasons overlap</h2>

Table 1 shows that Boro, Aus, and Aman overlap in several months. Therefore, the same insect observations may be included in more than one season. The Venn diagrams do not solve this problem and do not provide independent seasonal comparisons. The authors should use actual planting, crop-stage, and harvest records for each year and define non-overlapping analytical periods. If these records are unavailable, month or date should be analysed directly, and the general rice calendar should be used only for interpretation.

<h2>7. Weather effects are confounded with season</h2>

Temperature, rainfall, humidity, month, and rice season are closely related. The three temperature variables are also strongly correlated with each other. The authors should not use maximum, minimum, and mean temperature together without evaluating collinearity. They should choose biologically meaningful predictors, report VIF or another collinearity assessment, separate seasonal patterns from additional weather effects, and examine reasonable lagged effects of weather on insects.

<h2>8. Correlation is overinterpreted</h2>

A positive correlation between a pest and a predator does not prove a predator-prey relationship. Both groups may simply respond to the same month, weather, crop stage, or trapping conditions. A negative correlation does not prove pest suppression. The manuscript should replace words such as ‘influence’, ‘regulate’, ‘suppression’, ‘dependency’, and ‘biological control’ with ‘association’ unless stronger analysis supports a causal statement. Predator-prey relationships should be treated as exploratory hypotheses.

<h2>9. Light-trap catch is not the same as field population density</h2>

Light traps measure the activity and attraction of phototactic insects. Catch can change because of night length, rainfall during trapping, wind, cloud cover, moonlight, bulb condition, surrounding lights, and insect flight activity. The authors should use terms such as ‘light-trap catch’ or ‘activity-density’ rather than claiming that the trap directly measured the true field population. The statement that a light trap captures about 94% of the total field population is very doubtful and should be verified carefully or removed.

<h2>10. Crop and management information is missing</h2>

The paper links peaks to crop reproductive stages, but it does not provide actual yearly data on rice variety, planting date, crop stage, area planted around the trap, irrigation, insecticide application, fertilizer, harvest, fallow periods, or changes in nearby land use. These variables may strongly affect catches. The authors should provide these records. Without them, conclusions about crop stage and transitional periods must be weakened.

<h2>11. Taxonomic identification needs much more detail</h2>

Most taxa are presented only by common names or broad groups. The authors should provide scientific names where possible, identification keys, the names or qualifications of identifiers, treatment of damaged or uncertain specimens, and voucher deposition information. Broad groups such as spiders, carabids, staphylinids, and ladybird beetles include species with different ecological roles. Also, spiders are not insects; use ‘arthropod natural enemies’ instead of ‘beneficial insects’.

<h2>12. Figures and tables should be redesigned</h2>

Figure 2 should show months in calendar order from January to December. Figure 3 uses violin plots with very few data points and allows rainfall density below zero, which is impossible. Figures 4 and 5 use Venn diagrams for seasonal totals, but these figures hide time, effort, and uncertainty. Replace them with chronological time-series plots and model-based seasonal estimates with 95% confidence intervals. Every table should report units, n, variation, and the statistical model used.

<h2>13. The IPM conclusions are too strong</h2>

The study did not measure field infestation, crop damage, yield loss, economic thresholds, predator feeding, or the effect of an intervention. Therefore, it cannot demonstrate that natural enemies controlled pests or that insecticide use can be reduced. The results may help identify periods for closer local monitoring, but management recommendations must be presented as hypotheses that require field validation.

<h2>14. Data availability is inadequate</h2>

The statement that all data are presented in the manuscript is not correct because only aggregated results are shown. The authors should deposit the minimum date-level dataset, weather data, sampling-effort information, taxon dictionary, and analysis code in a public repository and provide a permanent link or DOI. ‘Data available on request’ is not sufficient for reproducibility.

<h2>15. Field permission and ethics information</h2>

The submission lists the ethics statement as not applicable, but the study involved field collection of insects. The authors should state that permission was obtained to operate the trap and collect insects at the BRRI site, name the responsible institution, and provide a permit number if one was required. If no permit was required, this should be stated clearly.

<h2>16. English and scientific writing</h2>

The manuscript contains many grammatical errors and unclear sentences in the abstract, introduction, methods, results, discussion, and conclusion. Terminology is also inconsistent. The paper requires complete English-language editing after the analysis is corrected. Use ‘insect pests’ instead of ‘harmful insects’, ‘abundance’ or ‘catch’ instead of ‘prevalence’, and ‘rainfall’ instead of ‘rain fall’.

<h1>**Recommended analysis** </h1>

·       Use trap-night as the preferred observation unit. If monthly data are used, retain the number of operational nights.

·       Analyse each taxon with a negative-binomial GLMM or GAMM. Test whether a zero-inflated model is needed.

·       Include an offset for trapping hours or operational nights.

·       Represent seasonality with month, a cyclic smooth, or clearly defined non-overlapping periods.

·       Include year as a random effect or temporal block and examine residual autocorrelation, such as AR(1).

·       Use a small set of biologically justified weather predictors and test collinearity.

·       Consider lagged weather effects and exploratory lagged pest-natural-enemy associations.

·       Correct for multiple testing across the many taxa and correlations.

·       Report effect estimates, 95% confidence intervals, diagnostics, and sensitivity analyses.

<h1>**Minor and editorial comments** </h1>

1.     Revise the title to identify the six-year, single-site light-trap design.

2.     The abstract should state the study site, sampling method, observation unit, model used, and key numerical results.

3.     The introduction should end with clear hypotheses or predictions rather than only general objectives.

4.     Line 55 contains an incomplete expression: ‘yield losses year-1’.

5.     Describe the weather station, instruments, recording frequency, data quality control, and missing weather values.

6.     Check whether ‘penicillium light trap’ is the correct trap name.

7.     Correct ‘net bug’ if ‘net bag’ was intended.

8.     Give the trap dimensions, bulb manufacturer or type, wavelength or spectral information if available, and replacement schedule.

9.     State how specimens were killed, preserved, identified, counted, and stored.

10.  State whether very large catches were subsampled and how counting accuracy was checked.

11.  Report the R version and package versions. RStudio is only the interface, not the statistical software itself.

12.  Use chronological month order in all figures and tables.

13.  Use ‘damselfly’ and ‘dragonfly’ consistently.

14.  Define every abbreviation in each table or figure legend.

15.  Check Table 7 carefully because some reported temperature ranges appear inconsistent.

16.  Correct the author-name inconsistency ‘Najmul/Nazmul’.

17.  Correct ‘Principle Scientific Officer’ to ‘Principal Scientific Officer’.

18.  Correct spelling errors in affiliations, including ‘University’.

19.  Check all references, DOI formats, journal names, and whether each reference actually supports the statement cited.

20.  Shorten the discussion and organize it around findings supported by the revised models.

21.  Add a clear limitations paragraph before the management implications.

22.  Rewrite the conclusion so that it applies only to the BRRI Gazipur light-trap series.

<h1>**Minimum changes required before reconsideration** </h1>

·       Provide the raw date-level data, sampling-effort records, weather data, and analysis code.

·       Clearly describe trap operation, missing nights, data aggregation, units, identification, and sample sizes.

·       Conduct a new repeated-measures count time-series analysis.

·       Remove the current overlapping seasonal analysis or replace it with valid non-overlapping crop records.

·       Rewrite all causal and predator-prey claims as associations unless supported by stronger analyses.

·       Redesign the figures and tables and report uncertainty.

·       Provide scientific names, identification methods, voucher details, and field permission information.

·       Deposit the dataset and code in a public repository.

·       Undertake full scientific English editing.

==============================

Please submit your revised manuscript by Aug 28 2026 11:59PM. If you will need more time than this to complete your revisions, please reply to this message or contact the journal office at plosone@plos.org. When you're ready to submit your revision, log on to https://www.editorialmanager.com/pone/ and select the 'Submissions Needing Revision' folder to locate your manuscript file.

- A letter that responds to each point raised by the academic editor and reviewer(s). You should upload this letter as a separate file labeled 'Response to Reviewers'.
- A marked-up copy of your manuscript that highlights changes made to the original version. You should upload this as a separate file labeled 'Revised Manuscript with Track Changes'.
- An unmarked version of your revised paper without tracked changes. You should upload this as a separate file labeled 'Manuscript'.

If you would like to make changes to your financial disclosure, please include your updated statement in your cover letter. Guidelines for resubmitting your figure files are available below the reviewer comments at the end of this letter.

If applicable, we recommend that you deposit your laboratory protocols in protocols.io to enhance the reproducibility of your results. Protocols.io assigns your protocol its own identifier (DOI) so that it can be cited independently in the future. For instructions see: https://journals.plos.org/plosone/s/submission-guidelines#loc-laboratory-protocols. Additionally, PLOS ONE offers an option for publishing peer-reviewed Lab Protocol articles, which describe protocols hosted on protocols.io. Read more information on sharing protocols at https://plos.org/protocols?utm_medium=editorial-email&utm_source=authorletters&utm_campaign=protocols.

As the corresponding author, your ORCID iD is verified in the submission system and will appear in the published article. PLOS supports the use of ORCID, and we encourage all coauthors to register for an ORCID iD and use it as well. Please encourage your coauthors to verify their ORCID iD within the submission system before final acceptance, as unverified ORCID iDs will not appear in the published article. *Only*  the individual author can complete the verification step; PLOS staff *cannot*  verify ORCID iDs on behalf of authors.

We look forward to receiving your revised manuscript.

Kind regards,

Mostafa Ghafouri Moghaddam, Ph.D

Academic Editor

PLOS One

Journal Requirements:

When submitting your revision, we need you to address these additional requirements.

1. Please ensure that your manuscript meets PLOS ONE's style requirements, including those for file naming. The PLOS ONE style templates can be found at

https://journals.plos.org/plosone/s/file?id=wjVg/PLOSOne_formatting_sample_main_body.pdf and

https://journals.plos.org/plosone/s/file?id=ba62/PLOSOne_formatting_sample_title_authors_affiliations.pdf

2. Please note that PLOS One has specific guidelines on code sharing for submissions in which author-generated code underpins the findings in the manuscript. In these cases, we expect all author-generated code to be made available without restrictions upon publication of the work. Please review our guidelines at https://journals.plos.org/plosone/s/materials-and-software-sharing#loc-sharing-code and ensure that your code is shared in a way that follows best practice and facilitates reproducibility and reuse.

3. Please ensure that you include a title page within your main document. We do appreciate that you have a title page document uploaded as a separate file, however, as per our author guidelines (http://journals.plos.org/plosone/s/submission-guidelines#loc-title-page) we do require this to be part of the manuscript file itself and not uploaded separately.

Could you therefore please include the title page into the beginning of your manuscript file itself, listing all authors and affiliations.

4. We note that Figure 1 in your submission contain map images which may be copyrighted. All PLOS content is published under the Creative Commons Attribution License (CC BY 4.0), which means that the manuscript, images, and Supporting Information files will be freely available online, and any third party is permitted to access, download, copy, distribute, and use these materials in any way, even commercially, with proper attribution. For these reasons, we cannot publish previously copyrighted maps or satellite images created using proprietary data, such as Google software (Google Maps, Street View, and Earth). For more information, see our copyright guidelines: http://journals.plos.org/plosone/s/licenses-and-copyright.

We require you to either (1) present written permission from the copyright holder to publish these figures specifically under the CC BY 4.0 license, or (2) remove the figures from your submission:

1. You may seek permission from the original copyright holder of Figure 1 to publish the content specifically under the CC BY 4.0 license.

We recommend that you contact the original copyright holder with the Content Permission Form (http://journals.plos.org/plosone/s/file?id=7c09/content-permission-form.pdf) and the following text:

“I request permission for the open-access journal PLOS ONE to publish XXX under the Creative Commons Attribution License (CCAL) CC BY 4.0 (http://creativecommons.org/licenses/by/4.0/). Please be aware that this license allows unrestricted use and distribution, even commercially, by third parties. Please reply and provide explicit written permission to publish XXX under a CC BY license and complete the attached form.”

Please upload the completed Content Permission Form or other proof of granted permissions as an "Other" file with your submission.

In the figure caption of the copyrighted figure, please include the following text: “Reprinted from [ref] under a CC BY license, with permission from [name of publisher], original copyright [original copyright year].”

2. If you are unable to obtain permission from the original copyright holder to publish these figures under the CC BY 4.0 license or if the copyright holder’s requirements are incompatible with the CC BY 4.0 license, please either i) remove the figure or ii) supply a replacement figure that complies with the CC BY 4.0 license. Please check copyright information on all replacement figures and update the figure caption with source information. If applicable, please specify in the figure caption text when a figure is similar but not identical to the original image and is therefore for illustrative purposes only.

The following resources for replacing copyrighted map figures may be helpful:

USGS National Map Viewer (public domain): http://viewer.nationalmap.gov/viewer/

The Gateway to Astronaut Photography of Earth (public domain): http://eol.jsc.nasa.gov/sseop/clickmap/

Maps at the CIA (public domain): https://www.cia.gov/library/publications/the-world-factbook/index.html and https://www.cia.gov/library/publications/cia-maps-publications/index.html

NASA Earth Observatory (public domain): http://earthobservatory.nasa.gov/

Landsat: http://landsat.visibleearth.nasa.gov/

USGS EROS (Earth Resources Observatory and Science (EROS) Center) (public domain): http://eros.usgs.gov/#

Natural Earth (public domain): http://www.naturalearthdata.com/

5. In the online submission form, you indicated that "All related data were presented in the manuscript. Data will be made available on request."

All PLOS journals now require all data underlying the findings described in their manuscript to be freely available to other researchers, either 1. In a public repository, 2. Within the manuscript itself, or 3. Uploaded as supplementary information.

This policy applies to all data except where public deposition would breach compliance with the protocol approved by your research ethics board. If your data cannot be made publicly available for ethical or legal reasons (e.g., public availability would compromise patient privacy), please explain your reasons on resubmission and your exemption request will be escalated for approval.

6. If the reviewer comments include a recommendation to cite specific previously published works, please review and evaluate these publications to determine whether they are relevant and should be cited. There is no requirement to cite these works unless the editor has indicated otherwise.

Additional Editor Comments :

Unfortunately, most expert reviewers declined to review your manuscript for several reasons. I carefully reviewed it, and before proceeding to the next step, which involves a great reviewer-by-reviewer process, you should consider my comments before anything happens.

My recommendation is major revision. The six-year dataset may support a useful local monitoring paper, but the current analysis and interpretation are not reliable enough for publication. Reconsideration should depend on access to the complete raw data, transparent reporting of sampling effort, and a full statistical reanalysis. If the authors cannot provide the raw data or correct the overlapping-season and time-series problems, rejection would be appropriate.

[Note: HTML markup is below. Please do not edit.]

Reviewers' comments:

[NOTE: If reviewer comments were submitted as an attachment file, they will be attached to this email and accessible via the submission site. Please log into your account, locate the manuscript record, and check for the action link "View Attachments". If this link does not appear, there are no attachment files.]

To ensure your figures meet our technical requirements, please review our figure guidelines: https://journals.plos.org/plosone/s/figures

You may also use PLOS’s free figure tool, NAAS, to help you prepare publication quality figures: https://journals.plos.org/plosone/s/figures#loc-tools-for-figure-preparation.

NAAS will assess whether your figures meet our technical requirements by comparing each figure against our figure specifications.

# Author comment: Author response to Decision Letter 1

**DOI:** 10.1371/journal.pone.0358423.r002

17 Aug 2026

Editor in Chief,

Thank you for your consideration and nice observation of our present manuscript. The author’s response according to the reviewer comments are present below, the correction (RED color) also made in the word file manuscript.

Reviewer comments and Author response

Major comments

1. Study design and scope

Only one light trap was used at one location. Repeated observations through time do not provide spatial replication. The title, abstract, discussion, and conclusion should clearly state that this is a single-site light-trap study in Gazipur. Broad statements about rice agroecosystems, Bangladesh, or tropical rice systems should be reduced.

Author Response: Thank you for your suggestion. We have revised the manuscript throughout to clearly define the scope of inference according to your suggestion. The study area has been explicitly described as a six year, single site light trap monitoring. We have changed the title also.

2. Sampling effort is not clear

The authors state that insects were collected daily, but they do not report the total number of operational trap nights, missing nights, power failures, bulb failures, maintenance periods, or other interruptions. The light operated for 10-12 hours, which means sampling effort was not always equal. The authors must report the number of trapping nights and hours for every sampling period and standardize the catches by effort or include effort as an offset in the model.

Author Response: Thank you for your valuable observation. We agree that a clear description of sampling effort is essential for interpreting long term light trap data. We have substantially revised these information including “light trap operation hour”, total trapping nights in our revised manuscript

3. Explain exactly how the data were summarized

It is not clear what the values in Tables 3-6 represent. They may be monthly totals, means per night, means of six yearly totals, or another measure. The authors must define the basic observation unit, show how daily catches were converted into monthly and seasonal values, report sample sizes, and give measures of variation such as standard error or confidence intervals.

Author Response: Thank you for your nice observations. In the revised manuscript, we now explicitly define the basic observational unit as the daily light trap catch (Number of individuals per operational trap night). Firstly, daily insect counts were recorded for each operational trap night then aggregated to obtain monthly mean catches per trap night. Monthly values were subsequently grouped according to the rice growing seasons. Standard error and co-efficient of variation have also been added to the revised tables to improve the tables.

4. The statistical analysis must be replaced

The response variables are insect counts. Count data are commonly non-normal, overdispersed, and sometimes zero-inflated. In addition, observations from consecutive days or months are not independent. A simple ANOVA with LSD comparisons and Pearson correlations is not sufficient. The authors should use a negative-binomial generalized linear mixed model or a generalized additive mixed model. The model should account for year, seasonality, unequal trapping effort, and temporal autocorrelation. Model assumptions and diagnostics must be reported.

Author Response: Thank you for this valuable comments. We have revised the statistical analysis for the monthly abundance of insect pests that explicitly account for over-dispersion. The daily trap night catch was the fundamental observational unit. For each insect taxon, abundance was analysed using a negative binomial generalized linear model (GLM). Details was included in our revised manuscript.

5. Monthly and seasonal tests are not described

The Methods section does not explain the tests that produced the P values, LSD values, and letter groupings in Tables 3-6. The authors must state the exact statistical test, model formula, post hoc method, software packages, sample size, and adjustment for multiple testing. Fisher's LSD across many months and many taxa can strongly increase false-positive results and should not be used without proper control.

Author Response: For monthly abundance we used negative binomial GLM in our revised manuscript. So, we have changed the table from Table 4 to Table 7 in our revised manuscript (Table 3 was newly added, that’s why table number has been changed). Thank you for your suggestions.

6. The rice seasons overlap

Table 1 shows that Boro, Aus, and Aman overlap in several months. Therefore, the same insect observations may be included in more than one season. The Venn diagrams do not solve this problem and do not provide independent seasonal comparisons. The authors should use actual planting, crop-stage, and harvest records for each year and define non-overlapping analytical periods. If these records are unavailable, month or date should be analysed directly, and the general rice calendar should be used only for interpretation.

Author Response: Thank you for your suggestion. We agree that the overlapping cultivation periods of Boro, Aus, and Aman rice in Bangladesh can result in temporal overlap in insect occurrence and that this issue needs to be distinguished from independent statistical comparisons among seasons. We have therefore revised the manuscript to clarify the analytical role of season, month, and the Venn diagrams. In the revised manuscript, seasonal abundance is presented separately according to the actual rice-growing calendar followed at the study site, and the Bangladesh rice calendar is used primarily to facilitate ecological interpretation of insect occurrence in relation to rice production periods. We acknowledge that the general rice calendar cannot, by itself, establish completely independent temporal periods when different rice crops are simultaneously present in the landscape. We used Venn diagram only for comparison of overlapping seasons and to show how season plays role for the transmission of insects transmitted from one season to another season. Because rice cultivation in Bangladesh involves overlapping cropping calendars, many insect species occur across multiple crop seasons. The Venn diagrams were specifically designed to visualize these ecological relationships rather than temporal abundance. Importantly, the Venn diagrams are not used as statistical tests of independent seasonal differences or as evidence of a causal “season effect.” Rather, they are retained as a complementary descriptive visualization to illustrate the shared and unique occurrence of insect taxa recorded during the Boro, Aus, and Aman production periods. Thus, the Venn diagrams address community overlap in taxonomic occurrence and should not be interpreted as resolving temporal pseudo-replication arising from overlapping rice seasons.

7. Weather effects are confounded with season

Temperature, rainfall, humidity, month, and rice season are closely related. The three temperature variables are also strongly correlated with each other. The authors should not use maximum, minimum, and mean temperature together without evaluating collinearity. They should choose biologically meaningful predictors, report VIF or another collinearity assessment, separate seasonal patterns from additional weather effects, and examine reasonable lagged effects of weather on insects.

Author Response: Thank you for your important suggestion. We would like to clarify, however, that the primary purpose of presenting the weather data in this manuscript was descriptive rather than predictive or causal. Specifically, the weather variables were summarized as monthly average meteorological conditions to provide environmental context for the observed seasonal and monthly patterns of rice insect abundance. They were not intended to constitute a multivariable predictive model in which maximum temperature, minimum temperature, mean temperature, rainfall, relative humidity, month, and season were simultaneously interpreted as independent explanatory variables.We acknowledge that a formal VIF/collinearity assessment and lagged modelling would be appropriate if the objective were to quantify the independent contribution of individual weather variables to insect abundance. However, such an analysis was not part of the original study design, and the available weather dataset was summarized at the monthly level

8. Correlation is over-interpreted

A positive correlation between a pest and a predator does not prove a predator-prey relationship. Both groups may simply respond to the same month, weather, crop stage, or trapping conditions. A negative correlation does not prove pest suppression. The manuscript should replace words such as 'influence', 'regulate', 'suppression', 'dependency', and 'biological control' with 'association' unless stronger analysis supports a causal statement. Predator-prey relationships should be treated as exploratory hypotheses.

Author Response: Thank you for your suggestions. You are alright. We have replaced the mention words from our revised manuscript. Also, improved correlation related section in both results and discussion where necessary.

9. Light-trap catch is not the same as field population density

Light traps measure the activity and attraction of phototactic insects. Catch can change because of night length, rainfall during trapping, wind, cloud cover, moonlight, bulb condition, surrounding lights, and insect flight activity. The authors should use terms such as 'light-trap catch' or 'activity-density' rather than claiming that the trap directly measured the true field population. The statement that a light trap captures about 94% of the total field population is very doubtful and should be verified carefully or removed.

Author Response: Thank you for your suggestion. We have removed the statements that a light trap captures about 94% of the total field population from the section 4.3. Implication of light trap for integrated pest management.

10. Crop and management information is missing

The paper links peaks to crop reproductive stages, but it does not provide actual yearly data on rice variety, planting date, crop stage, area planted around the trap, irrigation, insecticide application, fertilizer, harvest, fallow periods, or changes in nearby land use. These variables may strongly affect catches. The authors should provide these records. Without them, conclusions about crop stage and transitional periods must be weakened.

Author Response: Thank you for your suggestions. We have provided the rice season data in table 1. Although, rice variety, actual planting date, irrigation, fertilizer and insecticide data were missing, we have added some point regarding these in our revised manuscript in materials and methods section.

11. Taxonomic identification needs much more detail

Most taxa are presented only by common names or broad groups. The authors should provide scientific names where possible, identification keys, the names or qualifications of identifiers, treatment of damaged or uncertain specimens, and voucher deposition information. Broad groups such as spiders, carabids, staphylinids, and ladybird beetles include species with different ecological roles. Also, spiders are not insects; use 'arthropod natural enemies' instead of 'beneficial insects'.

Author Response: Thank you for your insightful comments. We have now provided scientific name with identifiers in our revised manuscript in Table 3. We have also used the term “arthropod natural enemy’ where applicable.

12. Figures and tables should be redesigned

Figure 2 should show months in calendar order from January to December. Figure 3 uses violin plots with very few data points and allows rainfall density below zero, which is impossible. Figures 4 and 5 use Venn diagrams for seasonal totals, but these figures hide time, effort, and uncertainty. Replace them with chronological time-series plots and model-based seasonal estimates with 95% confidence intervals. Every table should report units, n, variation, and the statistical model used.

Author Response: Thank you for your nice and insightful observations. There are six climatic seasons in Bangladesh, summer (from mid April to mid June), rainy (from mid June to mid August), autumn (from mid August to mid October), late autumn (from mid October to mid December), winter (from mid December to mid February) and spring (from mid February to mid April). These six seasons represent ecologically distinct climatic periods that strongly influence rice phenology, insect development, migration and natural enemy activity in Bangladesh. Therefore, organizing the data according to the national climatic seasons provide greater ecological relevance than chronological arrangement. We have changed the Figure 3 (for rainfall).

The purpose of Figure 4 and Figure 5 is not to describe temporal population dynamics but rather to summarize species occurrence and overlap among rice growing seasons (Aus, Aman and Boro). Unlike time series analyses, Venn diagrams provide an intuitive visualization of shared insect species among rice seasons; season specific species richness and ecological overlap of pest and beneficial arthropod communities. Because rice cultivation in Bangladesh involves overlapping cropping calendars, many insect species occur across multiple crop seasons. The Venn diagrams were specifically designed to visualize these ecological relationships rather than temporal abundance. Hopefully you considered.

13. The IPM conclusions are too strong

The study did not measure field infestation, crop damage, yield loss, economic thresholds, predator feeding, or the effect of an intervention. Therefore, it cannot demonstrate that natural enemies controlled pests or that insecticide use can be reduced. The results may help identify periods for closer local monitoring, but management recommendations must be presented as hypotheses that require field validation.

Author Response: Thank you for your comment. According to your suggestion, we have restructured our conclusion section.

14. Data availability is inadequate

The statement that all data are presented in the manuscript is not correct because only aggregated results are shown. The authors should deposit the minimum date-level dataset, weather data, sampling-effort information, taxon dictionary, and analysis code in a public repository and provide a permanent link or DOI. 'Data available on request' is not sufficient for reproducibility.

Author Response: The insect counts data, meteorological data used in this study and analytical code (R scripts) supporting the statistical analyses are not currently available in a public repository. However, all datasets and analytical code are available from the corresponding author upon reasonable request. This ststements has been added to the manuscript unser the data availability section.

15. Field permission and ethics information

The submission lists the ethics statement as not applicable, but the study involved field collection of insects. The authors should state that permission was obtained to operate the trap and collect insects at the BRRI site, name the responsible institution, and provide a permit number if one was required. If no permit was required, this should be stated clearly.

Author Response: Thank you for your comments. The authored are working under Bangladesh Rice Research Institute (BRRI). And the research field is belongs to BRRI, and this research was a part of regular monitoring of rice insect pest. So, no permission is required for light trap setting and insect collections.

16. English and scientific writing

The manuscript contains many grammatical errors and unclear sentences in the abstract, introduction, methods, results, discussion, and conclusion. Terminology is also inconsistent. The paper requires complete English-language editing after the analysis is corrected. Use 'insect pests' instead of 'harmful insects', 'abundance' or 'catch' instead of 'prevalence', and 'rainfall' instead of 'rain fall'.

Author Response: Thank you for your insightful suggestions. We have improved grammar and sentence throughout the revised manuscript. We also used suggested terminology.

Recommended analysis

· Use trap-night as t

Attachment Submitted filename: Author Response-P R1.docx

# Editor report: Decision Letter 1

**DOI:** 10.1371/journal.pone.0358423.r003

**Authors:** Mostafa Ghafouri Moghaddam

1 Sep 2026

Influence of weather variables on population dynamics of rice insect pests and their natural enemies: a six year light trap study in Gazipur, Bangladesh

PONE-D-26-19222R1

Dear Dr. Kumar Roy,

We’re pleased to inform you that your manuscript has been judged scientifically suitable for publication and will be formally accepted for publication once it meets all outstanding technical requirements.

Within one week, you’ll receive an e-mail detailing the required amendments. When these have been addressed, you’ll receive a formal acceptance letter and your manuscript will be scheduled for publication.

An invoice will be generated when your article is formally accepted. Please note, if your institution has a publishing partnership with PLOS and your article meets the relevant criteria, all or part of your publication costs will be covered. Please make sure your user information is up-to-date by logging into Editorial Manager at Editorial Manager® and clicking the ‘Update My Information' link at the top of the page. For questions related to billing, please contact billing support.

If your institution or institutions have a press office, please notify them about your upcoming paper to help maximize its impact. If they’ll be preparing press materials, please inform our press team as soon as possible -- no later than 48 hours after receiving the formal acceptance. Your manuscript will remain under strict press embargo until 2 pm Eastern Time on the date of publication. For more information, please contact onepress@plos.org.

Kind regards,

Mostafa Ghafouri Moghaddam, Ph.D

Academic Editor

PLOS One

Additional Editor Comments (optional):

The authors have thoroughly addressed the reviewers’ comments and substantially improved the manuscript. The revisions clarify the single-site light-trap design and scope of inference, strengthen the description of sampling effort and data aggregation, replace the previous statistical approach with appropriate negative-binomial GLMs (including model diagnostics and adjusted pairwise comparisons), refine taxonomic detail, and present more cautious interpretations of correlations and management implications. The English-language editing and improved consistency throughout the text are also appreciated. These changes satisfactorily resolve the major concerns raised during review, and the manuscript is now scientifically suitable for publication.

Reviewers' comments:

# Editor report: Acceptance letter

**DOI:** 10.1371/journal.pone.0358423.r004

**Authors:** Mostafa Ghafouri Moghaddam

PONE-D-26-19222R1

PLOS One

Dear Dr. Roy,

I'm pleased to inform you that your manuscript has been deemed suitable for publication in PLOS One. Congratulations! Your manuscript is now being handed over to our production team.

At this stage, our production department will prepare your paper for publication. This includes ensuring the following:

* All references, tables, and figures are properly cited

* All relevant supporting information is included in the manuscript submission,

* There are no issues that prevent the paper from being properly typeset

You will receive further instructions from the production team, including instructions on how to review your proof when it is ready. Please keep in mind that we are working through a large volume of accepted articles, so please give us a few days to review your paper and let you know the next and final steps.

Lastly, if your institution or institutions have a press office, please let them know about your upcoming paper now to help maximize its impact. If they'll be preparing press materials, please inform our press team within the next 48 hours. Your manuscript will remain under strict press embargo until 2 pm Eastern Time on the date of publication. For more information, please contact onepress@plos.org.

You will receive an invoice from PLOS for your publication fee after your manuscript has reached the completed accept phase. If you receive an email requesting payment before acceptance or for any other service, this may be a phishing scheme. Learn how to identify phishing emails and protect your accounts at https://explore.plos.org/phishing.

If we can help with anything else, please email us at customercare@plos.org.

Thank you for submitting your work to PLOS One and supporting open access.

Kind regards,

PLOS One Editorial Office Staff

on behalf of

Dr Mostafa Ghafouri Moghaddam

Academic Editor

PLOS One
